# *Clostridium sporogenes*-derived metabolites protect mice against colonic inflammation

**DOI:** 10.1080/19490976.2024.2412669

**Published:** 2024-10-14

**Authors:** Felix F. Krause, Kira I. Mangold, Anna-Lena Ruppert, Hanna Leister, Anne Hellhund-Zingel, Aleksandra Lopez Krol, Jelena Pesek, Bernhard Watzer, Sarah Winterberg, Hartmann Raifer, Kai Binder, Ralf Kinscherf, Alesia Walker, Wolfgang A. Nockher, R. Verena Taudte, Wilhelm Bertrams, Bernd Schmeck, Anja A. Kühl, Britta Siegmund, Rossana Romero, Maik Luu, Stephan Göttig, Isabelle Bekeredjian-Ding, Ulrich Steinhoff, Burkhard Schütz, Alexander Visekruna

**Affiliations:** a Institute for Medical Microbiology and Hygiene, Philipps-University, Marburg, Germany; b Institute of Anatomy and Cell Biology, Philipps-University, Marburg, Germany; c Core Facility for Metabolomics, Department of Medicine, Philipps-University, Marburg, Germany; d Flow Cytometry Core Facility, Philipps-University, Marburg, Germany; e Research Unit Analytical BioGeoChemistry, Helmholtz Zentrum, München, Germany; f Institute for Lung Research, Philipps-University, Marburg, Germany; g Department for Respiratory and Critical Care Medicine, Philipps-University, Marburg, Germany; h Member of the German Center for Lung Research (DZL/UGMLC, ) and German Center for Infectious Disease Research (DZIF), Marburg, Germany; i iPATH.Berlin, Core Unit of Charité-Universitätsmedizin Berlin, Charité - Universitätsmedizin Berlin, Corporate Member of Freie Universität Berlin, Humboldt-Universität zu Berlin, and Berlin Institute of Health, Berlin, Germany; j Department of Gastroenterology, Infectious Diseases and Rheumatology, Charité - Universitätsmedizin Berlin, Corporate Member of Freie Universität Berlin, Humboldt-Universität zu Berlin, Berlin, Germany; k Lehrstuhl für Zelluläre Immuntherapie, Medizinische Klinik und Poliklinik II, Universitätsklinikum Würzburg, Würzburg, Germany; l Institute of Medical Microbiology and Infection Control, Goethe University Frankfurt, University Hospital, Frankfurt am Main, Germany

**Keywords:** Colitis, commensal bacteria, indole-3-propionate, microbial metabolites

## Abstract

Gut microbiota-derived metabolites play a pivotal role in the maintenance of intestinal immune homeostasis. Here, we demonstrate that the human commensal *Clostridium sporogenes* possesses a specific metabolic fingerprint, consisting predominantly of the tryptophan catabolite indole-3-propionic acid (IPA), the branched-chain acids (BCFAs) isobutyrate and isovalerate and the short-chain fatty acids (SCFAs) acetate and propionate. Mono-colonization of germ-free mice with *C. sporogenes* (CS mice) affected colonic mucosal immune cell phenotypes, including up-regulation of *Il22* gene expression, and increased abundance of transcriptionally active colonic tuft cells and Foxp3^+^ regulatory T cells (Tregs). In DSS-induced colitis, conventional mice suffered severe inflammation accompanied by loss of colonic crypts. These symptoms were absent in CS mice. In conventional, but not CS mice, bulk RNAseq analysis of the colon revealed an increase in inflammatory and Th17-related gene signatures. *C. sporogenes*-derived IPA reduced IL-17A protein expression by suppressing mTOR activity and by altering ribosome-related pathways in Th17 cells. Additionally, BCFAs and SCFAs generated by *C. sporogenes* enhanced the activity of Tregs and increased the production of IL-22, which led to protection from colitis. Collectively, we identified *C. sporogenes* as a therapeutically relevant probiotic bacterium that might be employed in patients with inflammatory bowel disease (IBD).

## Introduction

The human intestine provides a natural habitat for a complex ecosystem of microorganisms that modulate various aspects of host homeostasis, including the maturation and functionality of the mucosal epithelium and the innate immune cell compartment.^1,2^ Commensal bacteria live in close association with the gut epithelium and the mucosal immune system. Yet, little is known about specific interactions between individual members of the intestinal microbiota with host cells. Recent pre-clinical and clinical studies suggest that commensal bacteria essentially contribute to the maintenance of intestinal immune homeostasis by regulating mucosal IgA production and differentiation of colonic Foxp3^+^ regulatory T cells (Tregs).^3,4^ Commensal microbiota not only modulate local immunity in the gut but also exert immunomodulatory effects on the systemic immune system by secreting metabolites that can enter the bloodstream and modulate immune responses in various organs.^5,6^ An imbalance or alteration in the gut microbiota composition and reduction of microbial diversity is closely related to intestinal barrier damage and the development of chronic inflammation and malignancies such as inflammatory bowel disease (IBD) and colorectal cancer, respectively^7–9^ IBD, including Crohn’s disease (CD) and ulcerative colitis (UC), is frequently accompanied by increased intestinal permeability and gut dysbiosis.^10^ The importance of commensal bacteria and microbiota-derived metabolites for host health is reflected in defective development of intestinal epithelium and lymphoid organs such as Peyer’s patches in germ-free (GF) mice, devoid of intestinal microbiota.^11^ These animals display reduced production of secretory IgA and impaired differentiation of intestinal Tregs and Th17 cells.^12–14^

Homeostatic production of IL-22 by Th17 cells, γδT cells and innate lymphoid cells (ILCs) beneath the epithelium is crucial for the intestinal barrier function and the production of antimicrobial peptides (AMPs).^15^ IL-22 is also known to support the restitution of goblet cells and mucus production leading to alleviation of colonic inflammation induced by dextran sodium sulfate (DSS).^16^ Similarly, a protective role for colonic tuft cells, which are able to enhance epithelial integrity, has been proposed in DSS-induced colitis.^17^ Although the underlying mechanisms leading to the alleviation of intestinal damage may be different, both, IL-22 production and activity of tuft cells appear to be regulated by microbial short-chain fatty acids (SCFAs),^18,19^ and by other small molecules synthesized by intestinal microbes such as succinate.^20–23^ The SCFAs acetate, propionate, and butyrate are the main group of microbial molecules generated by commensal bacteria through dietary fiber fermentation in the intestinal lumen.^24^ Besides SCFAs, several other gut microbiota-derived metabolites such as microbial tryptophan catabolites, secondary bile acids, and polyamines have been reported to promote beneficial and anti-inflammatory effects on the intestinal immune system.^25,26^ Thus, intestinal-resident bacteria generate a plethora of metabolites (some of those are still not characterized) that shape mucosal immunity and support intestinal homeostasis.

As the mucosal barrier and immune cells are crucial targets for commensals to exert their potential immunomodulatory activity, we were asking if specific bacteria are capable of producing metabolites that act on epithelial and immune cells in the lamina propria to mitigate colitis development. Here, we observed a significant immunomodulatory effect of the human commensal *Clostridium sporogenes* on mucosal cell populations and the local immune system. In contrast to GF and specific pathogen-free (SPF) animals, mice mono-colonized with *C. sporogenes* (CS mice) were completely protected from DSS-induced colitis. The beneficial influence of *C. sporogenes* on disease development was associated with a unique signature of microbial metabolites, which was not found in other commensals. In addition to generating high amounts of SCFAs and branched-chain fatty acids (BCFAs) like isobutyrate and isovalerate, *C. sporogenes* is one of the only a few gut bacteria currently known to synthesize 3-indole propionic acid (IPA) from dietary tryptophan.^27,28^ The microbial molecules IPA, SCFAs and BCFAs acted complementary in order to raise the numbers of colonic tuft cells, the production of IL-22, and the polarization of Foxp3^+^ Tregs, which led to complete protection from intestinal inflammation. In conclusion, we show that the human commensal *C. sporogenes* has a strong therapeutic potential by producing specific anti-inflammatory molecules being able to enhance epithelial barrier function and suppress pro-inflammatory immune responses in the gut.

## Materials and methods

### Mice

WT mice on a C57BL/6N background were purchased from Charles River, while ChAT-EGFP transgenic mice^29^ and Oligo-MM^12^ animals were bred in-house. *Ahr*^−/−^ mice were kindly provided by Prof. Dr. Ingo Schmitz (Ruhr-University, Bochum). All mice were kept under SPF conditions at the Biomedical Research Center, Philipps-University of Marburg. Eight-to-sixteen-week-old mice were used for experiments. GF breeding pairs with a C57B/6N background were generously provided by Dr. M. Basic from Hannover Medical School. These mice, along with gnotobiotic counterparts (mice mono-colonized with *C. sporogenes*), were bred in sterile isolators featuring positive pressure differential and filter-top cages. The isolators contained autoclaved bedding, food, and water provided *ad libitum*. Monthly sterility checks for GF mice involved culturing feces in a thioglycollate medium under both aerobic and anaerobic conditions for a minimum of 10 days, in addition to fecal gram staining. For gnotobiotic mice, contamination checks were performed by culturing feces on blood agar plates under aerobic conditions. All procedures involving GF and gnotobiotic mice were carried out within a laminar flow hood, ensuring sterile conditions. All animals were held following the guidelines established by the European Community for the care and use of animals. Breeding and use of samples of sacrificed mice for further experiments were approved by the responsible authorities at the Regional Council RP Giessen (Hesse), Germany, under approved protocols (G24/2019, Ex19-2019, G49/220,).

### *Colonisation of mice with* C. sporogenes

For colonization, *C. sporogenes* (ATCC 19404) was grown in BHI medium under anaerobic conditions for 1 day and washed in PBS. Then, 5 mL PBS containing *C. sporogenes* at an OD_600_ of 2 was once sprinkled on the food pellets of GF mice. Colonization and possible contamination was tested 2 weeks later, by plating out faces from colonized mice on blood agar plates. Species identification of colonies were then determined via MALDI-TOF-MS to confirm successful *C. sporogenes* colonization (Figure S1A).

### In vitro T cell differentiation

Mice aged between 8 and 16 weeks were sacrificed by cervical dislocation. Superficial cervical, deep cervical, axillary, brachial, renal, mesenteric, inguinal, lumbar, sacral and sciatic lymph nodes and spleen were isolated and single-cell suspensions were prepared by rubbing through a 30 µm filter. Cells were washed in BSS and centrifuged at 1500 rpm for 5 min at 4°C. The cells were then incubated in 4 mL NH_4_Cl for 5 min to lyse red blood cells. Cells from lymph nodes and spleen were pooled and resuspended in MACS buffer (Miltenyi Biotec). After counting, CD4^+^ T cells were purified using a negative CD4 isolation kit (Miltenyi Biotec) according to the manufacturer’s protocol. T cells were stained with anti-CD4 (RM4 5, PerCP, Biologend) and measured by flow cytometry. Samples with a purity of ≥95% were used for further experiments. Isolated T cells were transferred in RPMI media to 96- or 12-well plates at 2 × 10^6^ cells/mL (200 µl total volume in a 96-well plate, 2 ml volume in a 12-well plate) and subsequently stimulated with plate-bound anti-CD3 (5 µg/mL, BioLegend) and soluble anti-CD28 (1 µg/mL, BioLegend). Th17 were differentiated in the presence of anti-IFN-γ (5 µg/mL, BioLegend), anti-IL-4, IL-2 (25 U/mL), TGF-β1 (1 ng/mL, Peprotech) and IL-6 (60 ng/mL, Peprotech) for 3 days (37°C, 5% CO2). Tregs were polarized in the presence of anti-IFN-γ (5 µg/mL), anti-IL-4, IL-2 (100 U/mL) and TGF-β1 (3 ng/mL) for three days (37°C, 5% CO2). Stimuli were routinely added in a volume of 5 µl for a 96-well plate and 50 µL for a 12-well plate.

### Flow cytometry

Before flow cytometry analysis, T cells were harvested by pipetting up and down and restimulated with 50 ng/mL phorbol 12-myristate 13-acetate (PMA), 750 ng/mL ionomycin and 10 μg/mL brefeldin A (all by Sigma-Aldrich) in 1 ml RPMI medium in a 5 mL FACS tube for 4 h at 37°C, 5% CO2. Cells were then washed in PBS/FCS and single-cell suspensions were stained in 150 µL volume with anti-CD4 (RM4 5, PerCP) in 5 mL FACS tubes. After fixation with 2% formalin in PBS, cells were washed in PBS/FCS and permeabilised with saponin. Cells were stained in 150 µL saponin with 1:300 anti-IL-17A (12-7177-81), 1:500 anti-IFN-γ (17-7311-82), 1:300 anti-IL-22 (12-7221-82) or 1:300 anti-IL-13 (12-7133-81). All antibodies were purchased from BioLegend or eBioscience. Cells were subsequently washed in saponin, followed by PBS/FCS and then analyzed in the flow cytometer. For intracellular staining, cells were harvested and washed in PBS and then fixated with Foxp3 Fixation/Permeabilization Buffer Set (Thermo Fisher Scientific). Cells were then washed in PBS/FCS followed by saponin and then stained in 150 µL saponin with 1:300 anti-Foxp3 antibody (12-5773-82), 1:300 anti-IRF4 (646404) or 0.25 µg anti-RORγt (12-6981-82). Cells were then washed again in saponin, followed by PBS/FCS and then analyzed in the flow cytometer. For staining of phosphorylated proteins, cells were washed in cold PBS and fixated in 400 µL 2% formalin for 10 min at 37°C in 5 mL FACS tubes. After incubation, 3.6 mL −20°C cold methanol was added, and the cells were rested for 30 min on ice. Subsequently, the cells were washed in a wash buffer (PBS, 2% FCS, 0,2% Tween-20) and stained in a wash buffer for 1 h at RT. The following antibodies were used: 0.06 µg anti-p-mTOR (12-9718-42) and 0.06 µg anti-p-S6 (17-9007-42). Afterwards, the cells were washed again in a wash buffer, followed by PBS/FCS and then analyzed in the flow cytometer. All stained cells were examined using an Attune NxT Flow Cytometer (Thermo Fisher) with FSC 180 V, SSC 350 V, PE 320 V, PerCP 400 V, APC 360 V. Data were analyzed with FlowJo analysis software (TreeStar, v.10.8.1).

### Western blot

For immunoblotting, protein extracts were obtained by lysis of *in vitro* generated T cells in RIPA-Buffer. SDS-PAGE was performed following the measurement of protein concentration via BCA and denaturation in 6 × Laemmli buffer. Proteins were then transferred to a PVDF-membrane, blocked with 5% BSA in TBS buffer and incubated with primary antibodies in 5 mL 5% BSA in TBS buffer (1:1000 p-NF-κB p65 (3033S, CST), 1:1000 *p*-4E-BP1 (2855T, CST) overnight. The membrane was then treated with a secondary HRP-linked antibody (1:2000 7074S or 7076S, CST) and incubated for 2 h. For visualization, Western Blotting Luminol Reagent (Santa Cruz Biotechnology) was added and analyzed in a chemiluminescence imaging system. As loading control, β-actin (1:10000 A5441, Sigma-Aldrich) was used.

### RNA sequencing

For sequencing of T cells, total RNA was purified from *in vitro* generated, IPA-treated murine Th17 cells using the EXTRACT ME total RNA kit (BLIRT). For sequencing of colon tissue, mice were sacrificed, and the whole colon was isolated, opened longitudinal and cut into 1 cm long pieces. The tissue was homogenized in EXTRAzol with a hand blender (IKA® T10 basic, homogenizer) for 1 min. After complete homogenization, the samples were centrifuged at 12.000 g for 10 min at 4°C and the supernatant containing RNA was transferred to an RNase-free tube. Phase separation was performed by adding chloroform (200 µL/mL TRIzol). Following centrifugation at 12.000 g for 5 min at 4°C, the upper phase containing the RNA was transferred into a new tube and overlaid with isopropanol to precipitate the RNA. After centrifugation at 12.000 g for 10 min 4°C, the supernatant was discarded and the pellet was overlaid with 75% ethanol. After another centrifugation step, the pellet was dried and resuspended in RNA-free water. Concentrations were determined using a NanoDrop spectrophotometer. RNA from T cells and tissue were both treated with DNase (TURBO DNA-*free*™ Kit, Thermo Fisher Scientific) before sequencing. The purified RNA was quality-controlled by capillary gel electrophoresis and sequenced on an Illumina NextSeq 550 device. Reads were aligned to the genome of *Mus musculus* revision GRCm39 (mm39) with Qiagen CLC workbench v.10.0. Counts were calculated and normalized to one million mapped exonic reads (transcripts per million, TPM). Sets of differentially expressed genes were calculated in R (v. 4.3.0) with the DESeq2 package (v.1.40.2). Only genes with an adjusted p-value of <0.05 were considered as significantly regulated.

TPM values were computed, z-score transformed and represented in heatmaps. Heatmaps were generated with the R package pheatmap v. 1.0.12 using Euclidean distances. Cluster trees indicate hierarchical clustering. The KEGG Pathway Database was used for the pathway enrichment analysis. Sequencing data were deposited at NCBI GEO under accession numbers GSE193358 (T cells) and GSE264024 (colon tissue).

### Quantitative real-time polymerase chain reaction (qRT-pcr)

For qRT-PCR, total RNA was purified from *in vitro* generated, IPA-treated murine Th17 cells using the EXTRACT ME total RNA kit (BLIRT) with on column DNase treatment following the manufacturer’s protocol. Concentrations were determined using a NanoDrop spectrophotometer. cDNA synthesis was performed using the RevertAid First Strand cDNA Synthesis Kit (Thermo Fisher Scientific) according to the manufacturer’s instructions using a 500 ng template. qPCR was performed on a StepOne Plus instrument (Applied Biosystems). The Takyon ROX SYBR Master Mix blue dTTP kit (Eurogentec) was used for analysis. For mRNA quantification, mRNA expression was normalized to the housekeeping gene *HPRT* using the 2^−ΔΔCt^ method. The following murine primers were used: *HPRT* fwd 5′-CTG GTG AAA AGG ACC TCT CG-3′, *HPRT* rev 5′-TGA AGT ACT CAT TAT AGT CAA GGG CA-3′, *FoxP3* fwd 5′-TTC CCA TTC ACA TGG CAG GCT TCA-3′, *FoxP3* rev 5′-TGT TTG TGA GAC GTT GGA GG-3′, *IL22* fwd 5′-GCT TGA GGT GTC CAA CTT CCA G-3′, *IL22* rev 5′- ACT CCT CGG AAC AGT TTC TCC C-3′, *IL13* fwd 5′-TTG CTT GCC TTG GTG GTC TC −3′, *IL13* rev 5′-CCA TAC CAT GCT GCC GTT GC-3′.

### Induction of experimental colitis by dextran sodium sulfate (DSS)

Age- (9–12 weeks) and sex-matched (female or male) SPF, GF or gnotobiotic mice were used. DSS 2.5% (w/v) (35 000–50 000 kDa, MP Biomedicals) was administered via the drinking water for 5 days. Thereafter, the mice were switched to normal water and analyzed on day 7.

### Isolation of lamina propria T cells

Lamina propria T cells were isolated using the Lamina Propria Dissociation Kit (Miltenyi Biotec) according to the manufacturer’s instructions. In short, mice were sacrificed and the colon was isolated, opened longitudinal and cut into 1-cm-long pieces. Epithelial cells were washed off in a 20 mL DTT (1 mm) containing PBS/FCS under shaking for 20 min at 37°C. The sample was then placed onto a 100 µm cell strainer, and the tissue collected in the filter was again shaken in DTT containing PBS/FCS for 20 min at 37°C. The sample was again loaded onto a 100 µm cell strainer and the tissue was transferred into HBSS (w/o) containing 0.01 M HEPES and again shaken for 20 min at 37°C. Tissues were then transferred to a preheated digestion solution in C tubes (Miltenyi Biotec) and processed by gentleMACS Octo Dissociator (Miltenyi Biotec). The obtained cell suspension was filtered on a 100 μm cell strainer and washed with MACS buffer. To remove erythrocytes or dead cells, the cell suspension was then centrifuged (20 min for 2000 RPM at RT, without applying the brake) in a density gradient (40% percoll in RPMI top layer containing the cells and 70% percoll in RPMI bottom layer). Cells were then extracted out of the transition zone of the density gradient and counted using the TC20 automated cell counter (Bio-Rad). Following, the isolated cells were restimulated, stained and analyzed via flow cytometry as already described in the “Flow cytometry” paragraph.

### Cultivation of bacteria

Bacteria were either grown in thioglycollate or brain heart infusion (BHI) medium for liquid culture or in Columbia agar with 5% sheep blood by 37°C. For liquid culture, bacteria were inoculated at an OD_600_ of 0.05 and incubated for 72 h. All used anaerobic bacteria were aerotolerant and handled under normal atmospheric conditions. For cultivation, all anaerobic bacteria were grown in an anaerobic jar containing anaerobic atmosphere generation bags. The following strains were used in this publication: *C. sporogenes* (ATCC 19404, purchased from ATCC), *C. difficile* (ATCC 9689, purchased from ATCC), *C. butyricum* (629, strain collection of the medical microbiology UKGM), *C. perfringens* (15563/74, strain collection of the medical microbiology UKGM), *C. innocuum* (1891, strain collection of the medical microbiology UKGM), *B. fragilis* (2102, strain collection of the medical microbiology UKGM), *E. faecalis* (ATCC 29212, purchased from ATCC) and *E. coli* (ATCC 25922, purchased from ATCC).

### Detection of SCFA, BCFA and IPA by MS

To detect IPA, samples (8–18 mg feces, 50 µL serum or 10 µL culture medium) were mixed by vortexing with 350 µL water, 10 µL acidic acid, and 1 nmol IPA-d2 as internal standard. To extract the IPA, 350 µL diisopropyl ether was added and thoroughly mixed using an overhead shaker. After centrifugation, the organic phase was dried using a speed vac and subsequently vortex mixed with 20 µL *N-tert*-Butyldimethylsilyl-*N*-methyltrifluoroacetamide with 1% *tert*-butyldimethylchlorosilane (Merck AG Darmstadt). After a short centrifugation step, derivatization was done for 1 h at 60°C. All derivatization steps were done under a fume hood. Appropriate PPE (safety goggles and gloves) was worn at all times. Fig. S1A shows a typical chromatogram with the extracted ion traces of IPA and IPA-d2 and the structures of the tert-butyldimethylsilyl (TBDMS) derivatives. A GC-MS method was applied using an Agilent 7890A gas chromatograph with 5975C mass spectrometer. Samples (1 µL; split 1:10) were injected via an autosampler on an HP-5 MS column (30 m x 0.25 mm; film thickness 0.25 µm) and eluted with helium (1 mL min^−1^). The inlet was 250°C and the transfer line 310°C. The applied temperature gradient is described in Supplementary Table 1. The mass spectrometer was operated in positive electron impact ionization (EI) mode. Electron energy was 70 eV and electron current 34.6 µA. MS source and MS quad temperatures were set to 230°C and 150°C, respectively. Mass spectra were acquired with selected ion monitoring of IPA (m/z 246 and 303) and IPA-d2 (m/z 248 and 305) between 4 and 4.6 min. The dwell time was 20 ms for each ion and the electron multiplier voltage was set to 1247 V. For quantification a 6-point calibration curve was used (0.2–5 nmol). Data analysis was performed using Mass Hunter Quantitative Analysis (B.05.00).

The SCFA and BCFA concentrations in feces and serum samples were determined analogously to the method of Lotti *et al*.^30^ Briefly, 25–150 mg feces samples were mixed with 1 mL HPLC water and homogenized within 1 hour using an overhead shaker and 2–3 steel balls with a diameter of 3 mm. The samples were then centrifuged at 13,000 × g at 4°C for 30 min. 100 μL of the aqueous supernatant (alternatively 100 μL serum or culture medium samples) were mixed with deuterated internal standards (C2: 1 nmol, C3-C6: each 100 pmol) and vortex mixed with 200 μL of methyl tert-butyl ether. After centrifugation, 100 µL of the organic phase was transferred into a GC vial with an insert, and 2 µL of the sample was injected into an Agilent 7000 series GC-MS/MS. The chromatographic separation was carried out on an Agilent J&W Wax column (30 m × 0.25 mm; film thickness 0.25 µm) and eluted with helium (1.2 mL min^−1^). Inlet and transfer line were set to 250°C. The applied temperature gradient is described in Supplementary Table 2. The mass spectrometer was operated in XTR-EI mode. Electron current was 34.6 µA and the temperature of the MS source was set to 150°C. Mass spectra were acquired in multiple reaction monitoring mode (MRM) between 8.5 and 13.3 min. The transitions, corresponding collision energies and retention times are listed in Supplementary Table 3. The dwell time was 25 ms for each ion and the electron multiplier voltage was set to 2382 V. For quantification, a single-point calibration method was applied, using an appropriate deuterated internal standard for each analyte. Data analysis was performed using Mass Hunter Quantitative Analysis (B.09.00). Due to the high volatility of the analytes, all samples, solvents, and vials used were cooled with ice during processing. To avoid contamination of the ubiquitous SCFAs, fresh solvents were always used for each sample series and gloves were worn during the entire processing.

### Non-targeted metabolomics

In some experiments, polar metabolites were measured as described earlier.^31^ In brief, non-targeted metabolomics using HILIC UHPLC-MS/MS was applied for polar metabolites. An aliquot of methanol extract (50 µL) was evaporated (40°C, SpeedVac Concentrator, Savant SPD121P, ThermoFisher Scientific, Waltham, MA, United States) and reconstituted in an aqueous solution containing 75% ACN. Samples were analyzed by using an UHPLC system (ExionLC, Sciex LLC, Framingham, MA, USA) coupled to a quadrupole time-of-flight mass spectrometer (X500 QTOF MS, Sciex LLC, Framingham, MA, USA). Mass calibration of the MS was performed prior the analysis of the study by using the calibrant delivery system pump of the X500 QTOF MS (ESI Negative Calibration Solution). Additionally, after every fifth injection an automatic mass calibration of the X500 QTOF MS was conducted for the TOF MS and TOF MS/MS mode. The MS method was set to IDA (Information Dependent Acquisition). The parameters are summarized in Table 1 for the negative ionization IDA mode. Hydrophilic interaction liquid chromatography was conducted by using an iHILIC®- Fusion UHPLC column SS (100 × 2.1 mm, 1.8 µm, 100 Å, HILICON AB, Umea, Sweden). Chromatographic conditions are as follows: Eluent A consisted of 5 mmol/L NH_4_Ac (pH 4.6) in 95% ACN (pH 4.6) and eluent B of 25 mmol/L NH_4_Ac (pH 4.6) in 30% ACN. The LC-run started with 0.1% B and kept for 2 minutes, following by the increase of B to 99.9% over 7.5 minutes. The starting condition of 99.9% B was kept for 2 minutes and reversed to 0.1% B within 0.1 minutes and equilibrated for 4 minutes prior to the next injection. The run was finished after 12.1 minutes. The flow rate was set to 0.5 mL/min, the column temperature was set to 40°C and 5 µL of sample was injected on the column and the sample manager was set to 4°C. Weak and strong wash consisted of 95% ACN and 10% ACN. Data processing and analysis were carried out as follows: LC-MS raw data (.wiff2) were post-processed using GeneData Expressionist Refiner MS 13.5 (GeneData GmbH, Basel, Switzerland) including chemical noise subtraction, intensity cutoff filter, chromatographic peak picking, deisotoping and library MS search on MS1 level (precursor mass tolerance 0.005 Dalton). Data processing resulted in a data matrix containing clusters (*m/z* and retention time values) and observed maximum intensity values for each sample. Maximum intensity values were normalized to the wet cecal weight. Metabolite identification was done by matching experimental tandem MS spectra against spectral libraries, downloaded from MassBank of North America by using MS PepSearch (0.01 Dalton mass tolerance for Precursor and Fragment Search) or by running authentic standards within the study batch. Matches with dot product over 500 were kept for further analysis. Multiple matches were filtered for the best match. For identification levels, level 1 identification was included matching against a set of authentic standards, level 2 included metabolites that were identified by spectral libraries search and level 3 was set for metabolites solely based on MS1 annotation within a precursor mass tolerance of 0.005 Dalton.

### Histology staining and analysis

Histologic analyses were performed after staining tissue sections with hematoxylin and eosin, or with Alcian blue and nuclear red staining solutions. Examination of histomorphological changes after DSS treatment was performed as previously described.^32^ In brief, an additive score was given for infiltration of inflammatory cells and epithelial damage.

### In situ hybridization

For gene expression analyses on tissue sections, about 1-cm-long pieces of the colon were placed in the TissueTek compound and quickly frozen in isopentane. After initial storage at −70°C, 16-µm-thick tissue sections were cut with a cryostat, mounted on silanized glass slides, and subjected to an established *in situ* hybridization protocol.^19^ The generation of a template for the detection of *Egfp* transcripts has been described previously.^33^ DNA fragments from *Ffar2* (GeneBank acc. no. NM_146187.4, nt 924–1672, 749 bp), and *Ffar3* (GeneBank acc. no. NM_01033316.2, nt 441–1207, 767 bp) were amplified by PCR from mouse C57BL/6J ileum cDNA, subcloned into pGEM-T (Promega), the inserted sequence confirmed by double-stranded sequencing, and used as templates for the generation of digoxigenin-labeled (*Egfp*), or 35S-labeled (*Ffar2* and *Ffar3*) sense and anti-sense riboprobes.

### Immunohistochemistry

For the detection of protein distribution in tissue sections, about 1–2-cm-long pieces of colon, cecum, ileum, jejunum, and duodenum were placed for 24 h at room temperature in methacarn fixative containing 60% (v/v) methanol, 30% (v/v) chloroform, and 10% (v/v) glacial acetic acid. Afterwards, the tissues were washed in absolute ethanol, cleared with xylene, and embedded in paraffin. Longitudinal and transverse sections, 7 µm thick, were cut with a microtome and mounted on silanized glass slides. The immunohistochemical procedure was essentially performed as described previously.^34^ Primary antibodies included rabbit anti-Dclk1 (Abgent; AP7219b, 1:300 final dilution), rabbit anti-Pou2f3 (Sigma-Aldrich; 1:200 final dilution), rabbit anti-CD3 (Agilent Dako; A452, 1:4.000 final dilution). For the detection of antigen–antibody conjugates, a biotinylated donkey anti-rabbit IgG antibody (Dianova; 711-065-152, 1:200 final dilution) was used in combination with an avidin-biotin-peroxidase complex (Vectastain Elite ABC kit, Vector Laboratories). Immunoreactions were visualized by 8 min incubation in 3,3-diaminobenzidine (Sigma-Aldrich), enhanced by the addition of 0.08% ammonium nickel sulfate (Fluka). For a better visual localization of signals in the tissues, some sections were counterstained with either hematoxylin or nuclear red. Quantification of DCLK1, Pou2f3, or CD3 immunoreactive cells was performed as follows: For DCLK1 cell counts in the colon, and Pou2f3 cell counts in ileum, jejunum and duodenum, in transverse sections the length of the mucosa was measured along the lamina muscularis mucosae using the freehand tool of ImageJ (NIH). Then, immunoreactive cells were counted and expressed as cells/mm. In the jejunum and duodenum, only cells from the basal 200 µm (crypts and lower villi) were included, while in the ileum and colon the whole mucosa was investigated. For colonic Pou2f3 counts, in longitudinal and transverse sections the mucosal area was measured using ImageJ, and the immunoreactive cells were counted and expressed as cells/mm.^2^ For CD3 cell counts, the mucosal area was again used to investigate lymphocyte cell numbers. In all analyses, 3–4 sections per tissue. Separated by at least 35 µm, cells were counted in a blinded fashion, and mean values were calculated using Adobe Prism.

### Statistical analysis

Statistical analyses were performed using the one-way or two- ANOVA (Prism 8.0, GraphPad Prism). Results were expressed as the mean ± SD, and *p* values of <0.05 were considered significantly. Differential gene expression from RNA sequencing was computed as logarithmic fold change with the DeSeq2 Package v. 1.40.2 in R v4.3.0. Genes were considered differentially expressed at padj. <0.05 (p-value adjusted by Benjamini – Hochberg correction for multiple testing).

## Results

### Clostridium sporogenes generates high amounts of tryptophan-derived IPA, SCFAs and BCFAs in vivo

**Figure 1.**
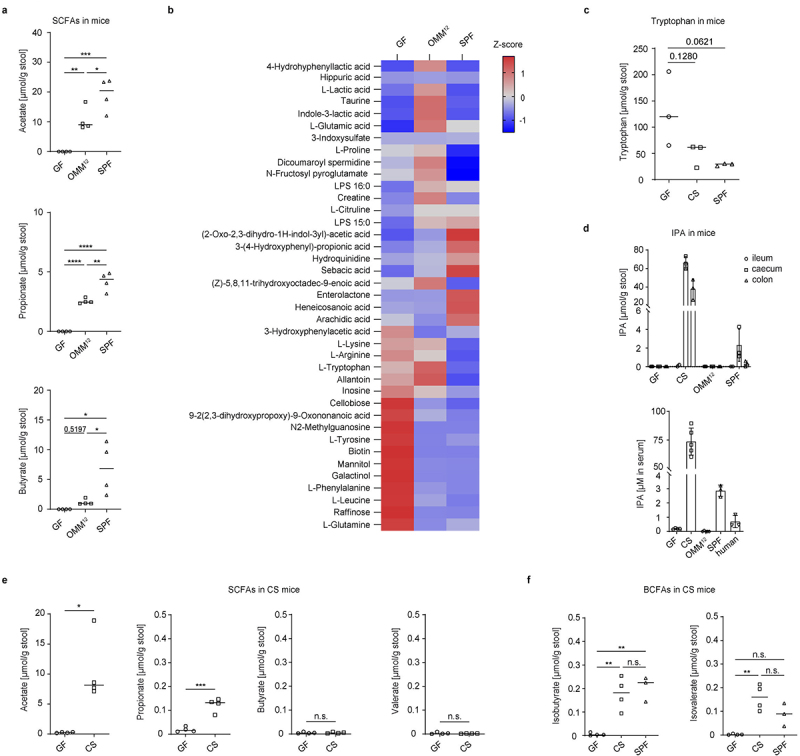
*C. sporogenes* synthesizes a broad range of metabolites in the gut of mice. (a) Levels of acetate, propionate, butyrate (*n* = 4) and (b) numerous small polar molecules in the caecum of GF, OMM^12^ and SPF mice, measured by GC-MS and LC-MS. (c) Levels of tryptophan in the colon of GF, CS and SPF mice measured by GC-MS (*n* = 3). (d) Detection of indole-3-propionic-acid (IPA) in GF, CS, OMM^12^ and SPF mice in the ileum (*n* = 2), caecum, (*n* = 3) colon (*n* = 3) and serum (*n* ≥3). Pooled human serum samples were measured as a reference (*n* = 3). (e) Levels of SCFAs and (f) BCFAs in the colon of GF and CS mice measured by GC-MS (*n* = 4). Statistical analysis was done with one-way ANOVA with *p < 0.05; **p < 0.01; ***p < 0.001; ****p < 0.0001. Data are presented as mean and error bars represent SD.

The microbiota-host communication is mediated by the bidirectional metabolic exchange of essential and non-essential metabolites.^35^ We used mass spectrometry (MS) analysis to compare the intestinal metabolic signatures of germ-free (GF) mice with mice harboring a simplified microbial community composed of 12 defined commensals (Oligo-MM,^12^ and with conventional animals harboring a complex gut microbiome (SPF mice). SCFAs are the major group of commensal metabolites generated by bacterial fermentation of complex carbohydrates in the gastrointestinal tract.^36^ We observed a gradual microbe-dependent increase in abundance of acetate, propionate and butyrate in the cecal content. All three SCFAs were not detectable in the cecum of GF mice, were present in higher abundance in Oligo-MM^12^ animals and reached a peak in SPF mice (Figure 1(a)). In addition, the abundance of numerous small polar molecules was significantly different between the three groups of mice (Figure 1(b)). Dietary amino acids including lysine, arginine, tryptophan, tyrosine, phenylalanine, leucine, and glutamine were highly enriched in the cecum of GF mice, indicating microbiome-dependent utilization of these molecules. In contrast, GF animals had lower levels of glutamic acid, which is a by-product of the shikimate pathway used by bacteria, suggesting a microbiome-induced accumulation of this amino acid. Interestingly, while the set of dietary amino acids was similarly utilized by minimal and complex microbiota, the abundance of free tryptophan was reduced only in the presence of the complex microbial community of SPF mice, but not by the 12 defined commensals (Figure 1(b)). Tryptophan is an essential amino acid that is involved in several physiological processes in the body including regulation of the immune system and neuronal functions, contributing to the health of the host.^26^ Because the complex metabolic networks within the microbial community make it challenging to identify commensals being capable of directly impacting the host immune system via tryptophan-derived microbial metabolites, we mono-colonized GF animals with *Clostridium sporogenes*, a commensal well-known for its efficient tryptophan catabolism.^27^ *C. sporogenes* is one of a few bacteria present in the human gut that is able to produce high amounts of tryptophan derivate indole-3-propionic acid (IPA) by reductive Stickland metabolism.^37^ We validated the successful mono-colonization by bacterial culture and MALDI-TOF MS analysis (Figure S1B). We observed a significant shrinkage of the cecum in mono-colonized (CS mice) compared to GF animals that are characterized by 2- to 4-fold enlarged cecum probably due to accumulation of undigested fibers and oligosaccharides (Figure S1C). Notably, the tryptophan levels measured in the cecal content were reduced in CS mice in comparison to GF animals, indicating that dietary tryptophan was efficiently metabolized by *C. sporogenes* (Figure 1(c)). Measurement of IPA production in GF, CS, Oligo-MM^12^ and SPF mice revealed a high amount of this microbial metabolite in the cecal and colonic stool of CS mice only, compared to a relatively low IPA production in SPF mice, and no IPA in Oligo-MM^12^ and GF animals (Figure 1(d)). Similar IPA amounts were found in serum, with high levels in CS, moderate production in SPF mice and human serum, and absence in GF and Oligo-MM^12^ mice (Figure 1(d)). Moreover, we observed that *C. sporogenes* grown in BHI medium generated all three common SCFAs acetate, propionate, and butyrate. Interestingly, the amount of the BCFAs isobutyrate and isovalerate was almost as high as that of SCFAs (Figure S1D). Next, we tested if the *in vivo* production of SCFAs and BCFAs by *C. sporogenes* was also detectable in the colon of CS mice. The amounts of the SCFAs acetate and propionate were strongly increased as compared to negligible levels in GF mice, while levels of butyrate and valerate did not increase (Figure 1(e)). Of note, we observed unusual accumulation of the two BCFAs isobutyrate and isovalerate in CS mice. Interestingly, the total production of BCFAs in the colonic content of CS mice was similar to SPF animals (Figure 1(f)). Collectively, these findings indicate that one specific commensal bacterium can generate high amounts of intestinal and circulating metabolites such as IPA, SCFAs, and BCFAs (Figure S1D).

### C. sporogenes-derived metabolites influence the mucosal immune system and intestinal tuft cells

**Figure 2.**
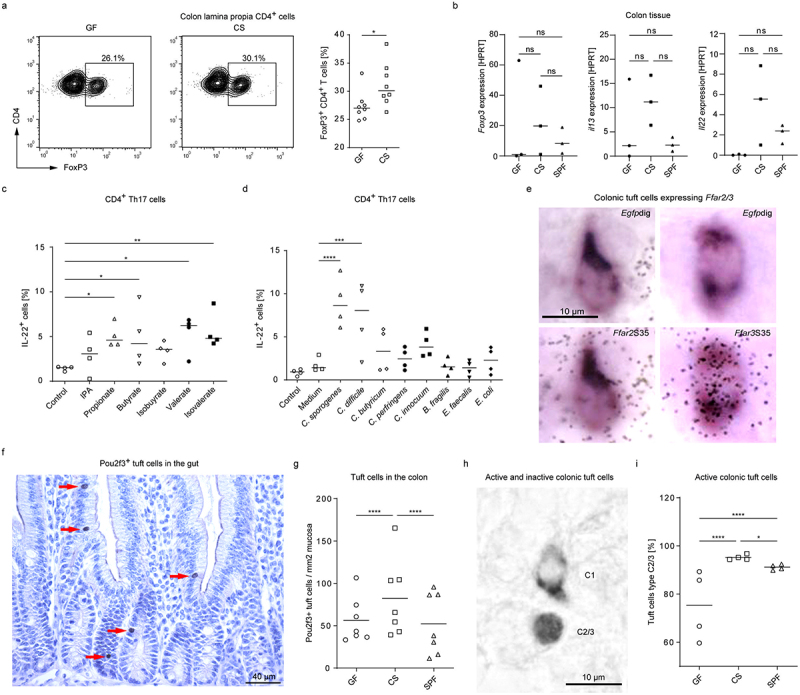
Alterations in the local immune system and colonic tuft cell abundance in mice mono-colonized with *C. sporogenes*. (a) Representative flow cytometry dot-blots and scatter blot of lamina propria FoxP3^+^ CD4^+^ T cells in the colon of GF and *C. sporogenes* mono-colonized (CS) mice (*n* = 8). (b) Bar graphs, showing the relative fold change of *Foxp3*, *Il13*, and *Il22* expression in the colon of GF, CS and SPF mice via quantitative real time PCR (qPCR). Expression is shown relative to hypoxanthine-guanine phosphoribosyltransferase (HPRT) expression (*n* = 3). (c,d) flow cytometry analysis of Th17 cells showing the percentage of IL-22^+^ cells after 3 days. CD4^+^ T cells were isolated from two to four month-old C57BL/6N mice and differentiated under Th17-polarising conditions for 3 days. (c) Cells were treated with *C. sporogenes* metabolites (IPA 50 µm; propionate, isobutyrate, valerate, isovalerate, each 2 mm; butyrate 0.5 mm) or (d) bacterial cell-free supernatants from day 0 on. (*n* ≥3). Statistical analysis was done with one-way ANOVA with *p < 0.05; **p < 0.01; ***p < 0.001; ****p < 0.0001. Data are presented as mean. (e) In double-labeling ISH experiments, colonic tuft cells (*Egfp*_dig_-labeled in chat-egfp mice) were found *Ffar2* negative, but *Ffar3* co-positive (representative images of *n* ≥100 analyzed cells). (f) Representative detection of Pou2f3^+^ tuft cells (labeled by red arrows) by immunohistochemistry in the gut of mice. (g) The density of colonic epithelial tuft cells was analyzed in GF, CS, and SPF mice by Pou2f3 immunohistochemistry (*n* = 7). Each point represents the mean of 3 single measurements. (h) Pou2f32f3 immunoreactivity discriminated C1-type (cytoplasmic signals) from C2/3-type (nuclear signals) colonic tuft cells. (i) Quantification of C2/3-type tuft cells in the colon of GF, CS, and SPF mice (*n* = 4). Statistical analysis was done with two-way ANOVA with Tukey post hoc test with *p < 0.05, ****p < 0.0001.

In addition to acting on the gut epithelium, specific metabolites produced by *C. sporogenes* are likely to be able to diffuse into the lamina propria and reach the immune compartment. Thus, we investigated whether CS mice exhibited alterations in the intestinal immune system in comparison to GF and SPF mice. When comparing the colonic lymphocytes in CS mice with that of GF animals, we detected an increased frequency of Foxp3^+^ Tregs and *Foxp3* expression in colonic lamina propria of mono-colonized mice (Figures 2(a,b)). Moreover, the mRNA expression of *Il22* and *Il13* was elevated in the colon of CS mice and was even higher than in SPF mice (Figure 2(b)). Interestingly, we observed that some SCFAs and BCFAs, but not IPA, induced the secretion of IL-22 by Th17 cells, suggesting a specific role for these microbial metabolites in regulating the function of IL-22-producing immune cells (Figure 2(c)). The same effect was seen for *C. sporogenes*- and *C. difficile*-derived supernatants, but not for other bacteria tested (Figure 2(d)).

Tuft cells are a minor epithelial chemosensory cell subtype that regulates e.g. type 2 immunity in the gut.^38^ Since SCFAs act as direct activators of some mucosa-associated tuft cells,^19^ and intestinal ILC2-derived IL-13 induces the differentiation of this cell type by activating crypt-based Lgr5-positive progenitor cells,^39^ we asked whether *C. sporogenes* mono-colonization influenced tuft cell abundance and activity under homeostatic conditions. Using *in situ* hybridization (ISH) to detect gene transcripts in tissue sections, we observed that the two SCFA receptors, *Ffar2* and *Ffar3*, were differentially expressed in the mouse colonic epithelium (Figure S2A). Expression of *Ffar2* was seen in many epithelial cells and most prominent in the lower crypt half. Expression of *Ffar3*, on the other hand, was restricted to single epithelial cells. As control of specificity, the use of sense riboprobes did not result in specific labeling (Figure S2B, C). Double-labeling ISH in mice expressing enhanced green fluorescent protein (EGFP) under the choline acetyltransferase (ChAT) promoter revealed *Ffar3*-expressing cells being cholinergic tuft cells, while *Ffar2* expression spared this cell type (Figure 2(e)), indicating that tuft cells are a specific SCFA target. The abundance of colonic tuft cells under a specific microbiome was determined using Pou2f3 immunoreactivity. Pou2f3 is an essential transcription factor needed for the development of tuft cells.^40^ Compared to GF and SPF mice, CS mice showed elevated colonic tuft cell numbers (Figure 2(f), (g), and Figure S2D-F). This was also the case for tuft cells in the ileum, jejunum and duodenum, although the effect was less prominent with further distance to the colon (Figure S2G-I). Based mainly on scRNA-seq analyses, intestinal tuft cells are a heterogeneous population that manifests in a neuronal-type (tuft-1) and an immune-type (tuft-2) phenotype.^39^ Recently, a third colonic tuft cell sub-cluster (named C1) has been proposed, which appears to be transcriptionally inactive, and mice may be more prone to impaired regeneration during acute colitis if this subtype predominates.^41^ Pou2f3 immunoreactivity was found as a marker to discriminate between transcriptionally inactive (C1, cytoplasmic signals) and transcriptionally active (C2/3, resembling tuft-1 and tuft-2 phenotypes, nuclear signals) tuft cells (Figure 2(h)). Notably, CS mice had the highest C2/3 counts when compared to GF and SPF mice (Figure 2(i)), suggestive of a superior reactivity when challenged.

#### C.sporogenes ameliorates colitis in mice by modulating the intestinal immune system

**Figure 3.**
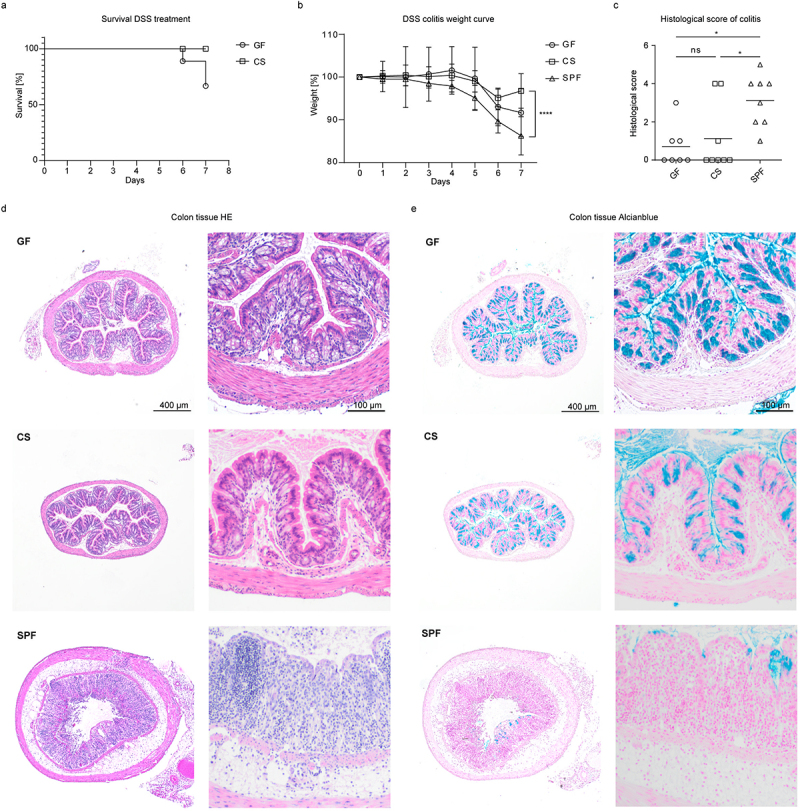
*C. sporogenes* protects mice against dss-induced colitis. (a) Survival, (b) weight curve and (c) histopathology scores of GF, CS and SPF mice treated with DSS (2.5%) in drinking water for 5 days (*n* ≥7). Statistical analysis was done with one-way ANOVA (a) and two-way ANOVA (c) with *p < 0.05, ****p < 0.0001. The horizontal line represents the mean. (d, e) Representative colon tissue sections from DSS-treated GF, CS and SPF mice stained with (d) hematoxylin and eosin (HE) or (e) Alcian blue and nuclear red (*n* = ≥ 7).

**Figure 4.**
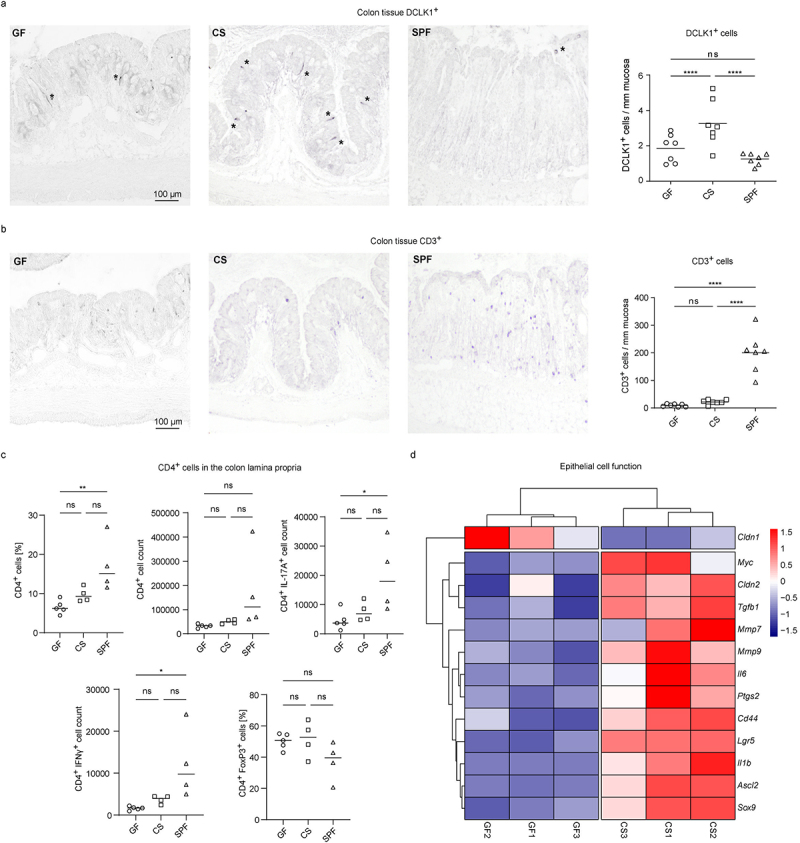
Analysis of epithelial and T cells in the colon of GF, CS and SPF mice. (a-d) GF, CS and SPF animals were orally treated with DSS (2.5%) in drinking water for 5 days and analyzed on day 7. (a,b) Representative immunohistochemistry staining of colon tissue and scatter plot from DSS-treated GF, CS and SPF mice stained with (a) anti-Dclk1 or (b) anti-CD3 antibodies (*n* = 7). Statistical analysis was done with two-way ANOVA with Tukey post hoc test with ****p < 0.0001. The horizontal line represents the mean. (c) Scatter plots showing CD4^+^ cells in the lamina propria of the colon in DSS-treated GF, CS and SPF mice. Shown are the percentage and cell count of CD4^+^ cells, cell count of IL-17A^+^ CD4^+^ and IFNγ^+^ CD4^+^ cells and the percentage of FoxP3^+^ CD4^+^ cells (*n* = 4-5). Statistical analysis was done with one-way ANOVA with *p < 0.05; **p < 0.01. The horizontal line represents the mean. (d) Heat map displaying significantly changed genes related to epithelial cell function the between GF and CS mice after DSS-induced colitis. Colon tissue was harvested, and RNA was extracted and sequenced (*n* = 3). Expression changes of genes were considered significant with p adjusted value < 0.05. Transcripts per million (TPM) values were computed, z-score transformed and represented in heatmaps. Heatmaps were generated with the R package pheatmap v. 1.0.12 using Euclidean distances. Trees indicate hierarchical clustering (complete method).

Colonic tuft cells, Tregs, and the cytokine IL-22, which were modulated by colonization of mice with *C. sporogenes*, have been described to be protective in experimental colitis models.^16,42,43^ We thus wondered if mice mono-colonized with *C. sporogenes* were prone to developing DSS-induced intestinal inflammation. As previously reported,^44^ GF mice were susceptible to epithelial injury in DSS-induced colitis, which resulted in high mortality within 6 days (Figure 3(a)). Notably, the colonization with *C. sporogenes* significantly increased the survival of animals following challenge with DSS (Figure 3(a)). SPF animals showed a high histopathology score, associated with increased weight loss and alterations in the crypt structure. In contrast, no clear signs of colonic inflammation were observed in CS mice that quickly regained weight after cessation of the stimulus (Figure 3(b-d)). Furthermore, Alcian blue staining revealed depletion of crypts and goblet cells in SPF mice, but not in GF and CS mice (Figure 3(e)). Similar to untreated mice (Figure 2(g)), the numbers of DCLK^+^ tuft cells were decreased in conventional mice treated with DSS as compared to mono-colonized animals (Figure 4(a)). Moreover, we detected a massive infiltration of CD3^+^ T lymphocytes only in the colonic mucosal lamina propria of SPF mice following the induction of acute inflammation by DSS (Figure 4(b)). FACS analysis confirmed these data, revealing a high number of pro-inflammatory IL-17A^+^ and IFN-γ^+^ CD4^+^ T cells in the colonic lamina propria of SPF, but not in that of CS mice (Figure 4(c)). These observations further demonstrated beneficial effects of *C. sporogenes* on DSS-induced colitis.

**Figure 5.**
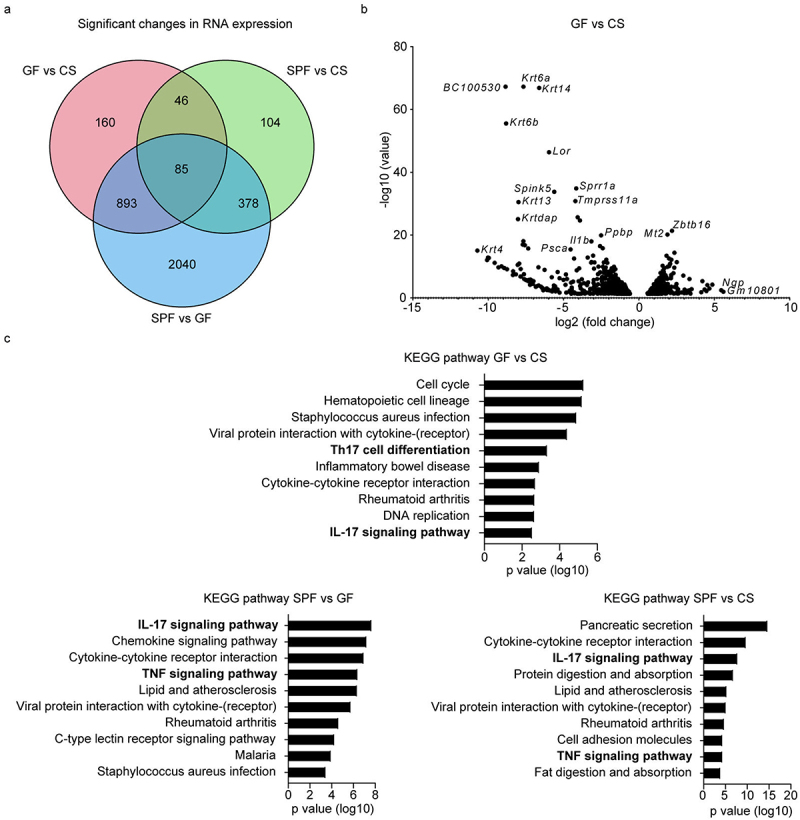
*C. sporogenes* influences Th17 cells in DSS-induced colitis. (a-c) GF, CS and SPF animals were orally treated with DSS (2.5%) in drinking water for 5 days and analyzed on day 7. Colon tissue was harvested, and RNA was extracted and sequenced (*n* = 3). Expression changes of genes were considered significant with *p* adjusted value < 0.05). (a) Venn diagram indicating the amount of significantly changed genes between the compared groups. Number of genes only changed in the comparison GF vs CS are red, SPF vs CS green and SPF vs GF blue. (b) Volcano plot of murine colon tissue after DSS treatment showing differently regulated genes between GF and CS mice. (c) KEGG pathway analysis showing the top 10 most changed pathways between SPF, CS, and GF mice.

To better understand the alterations in the intestinal immune compartment during the oral administration of DSS, we subjected colonic tissue from GF, CS, and SPF mice to RNA sequencing (RNA-seq). Notably, genes associated with epithelial cell function, stemness, self-renewal and differentiation were upregulated in CS mice as compared to GF animals (Figure 4(d)). Furthermore, the transcriptome analysis revealed significant differences in gene expression profiles between CS, SPF and GF animals after DSS exposure (Figure 5 (a), (b)). As compared to GF mice, CS animals had significantly upregulated genes involved in cell cycle regulation (Figure S3A). In contrast to CS mice, various genes implicated in the onset of intestinal inflammation and migration of immune cells to inflammatory sites such as alarmins *S100A8* and *S100A9* and pro-inflammatory chemokines *CCL3-5*, *CXCL1*, *CXCL5*, *CXCL9* and *CXCL13* were highly enriched in the SPF cohort of mice (Figure S3B). Among the pro-inflammatory factors, several genes associated with induction of Th17 cell differentiation (*IL1R1*, *IL1R2*, *IL1B*, *IL17RA* and *IL6*) were substantially enriched in SPF mice as compared to CS animals (Figure S3B). Finally, the analysis of KEGG-pathways confirmed significant changes in cytokine–cytokine receptor interactions, as well as in the TNF and IL-17 signaling pathways when we compared all three groups of mice (Figure 5(c)).

#### C. sporogenes-derived metabolites suppress differentiation of Th17 cells

**Figure 6.**
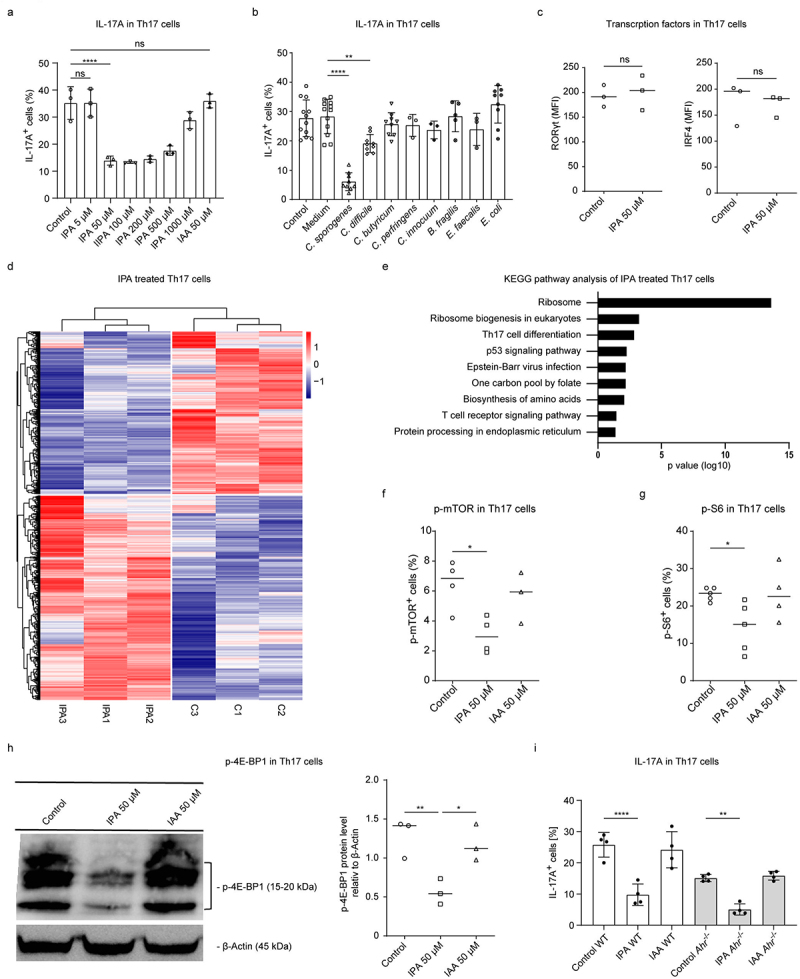
IPA reduces amounts of IL-17A in Th17 cells. (a-i) CD4^+^ T cells were isolated from two to four month-old C57BL/6N mice and differentiated under Th17-polarising conditions for 2 days for RNA-based and 3 days for protein-based methods. Cells were treated with different stimuli starting at day 0. (a) Flow cytometry analysis of Th17 cells showing percentage of IL-17A^+^ cells after 3 days (*n* = 3). (b) Flow cytometry analysis of Th17 cells showing the percentage of IL-17A^+^ cells after 3 days. Cells were treated with 2.5 vol% bacterial cell-free supernatant set to an OD_600_ of 0.7 (*n* ≥3). (c) Staining of RORγt and IRF4 in Th17 cells via flow cytometry on day 3 (*n* = 3). (d) Heatmap displaying all significantly (*p*adj. <0.05) changed genes between untreated and ipa-treated Th17 cells. Trees indicate hierarchical clustering (complete method). (e) KEGG pathway analysis showing the top 10 most changed pathways in ipa-treated Th17 cells compared to the control. (f) Phospho-staining of p-mTOR in Th17 cells via flow cytometry on day 3 of the cell culture (*n* ≥3). (g) Phospho-staining of p-S6 in Th17 cells via flow cytometry on day 3 of differentiation (*n* = 4). (h) Representative western blot of phosphorylated-4E-BP1 in Th17 cells and bar graphs showing relative *p*-4E-BP1 signals normalized to β-actin (*n* = 3). (i) Flow cytometry analysis of Th17 cells purified from WT (white) and *Ahr*^−/−^ (grey) mice showing the percentage of IL-17A^+^ cells after 3 days differentiation (*n* = 4). All statistical analyses were done with one-way ANOVA with *p < 0.05; **p < 0.01; ****p < 0.0001. Data are presented as mean and error bars represent SD.

Since the KEGG pathway analysis showed significant changes in the regulation of the IL-17 signaling pathway during colitis development between all three groups of mice, we examined the influence of *C. sporogenes*-derived metabolites on the differentiation of Th17 cells. As previously shown,^45^ SCFAs are capable of reducing the IL-17A expression in Th17 cells (Figure S4A). Next, we asked whether the *C. sporogenes*-specific metabolite IPA has the ability to modulate the production of IL-17A by Th17 cells. IPA is an end-product of the reductive Stickland metabolic pathway of dietary tryptophan, uniquely used by *C. sporogenes*.^37^ Interestingly, only IPA, but not its precursor molecule indole-3-acetic acid (IAA), was able to suppress the IL-17A synthesis in Th17 cells (Figure 6(a)). Similarly, the supernatant from *C. sporogenes* but not that of other *Clostridia* species (except *C. difficile* to a lesser extent), *Escherichia coli* or *Bacteroides fragiles*, strongly reduced the frequency of IL-17A in CD4^+^ T cells (Figure 6(b)). Intriguingly, when we measured the expression of transcription factors RORγt and IRF4 that are critical for Th17 differentiation and IL-17A production, we did not observe any differences between unstimulated and IPA-stimulated Th17 lymphocytes (Figure 6(c)). In addition, the activity of NF-кB, which is also known to be implicated in the generation of Th17 lymphocytes,^46^ was not affected by IPA treatment (Figure S4B). Furthermore, the IPA did not influence the proliferation of Th17 cells (Figure S4C). These observations led us to conclude that IPA may post-transcriptionally regulate the function of Th17 cells. A global RNA-seq analysis revealed strong alterations in the gene expression of IPA-treated Th17 cells as compared to control Th17 lymphocytes (Figure 6(d)). In addition, the KEGG-pathway analyses showed that the expression of several genes related to the ribosome and ribosome biogenesis was significantly altered following IPA treatment (Figure 6(e), Figure S4D). Since the mTOR complex, a central regulator of cellular metabolism, also directly regulates ribosomal protein synthesis by stimulating the phosphorylation of S6 kinase and 4E-BP1 protein, we investigated the influence of IPA on the mTOR signaling pathway. We found that treatment of Th17 lymphocytes with IPA results in reduced mTOR activity and decreased p-S6 and *p*-4E-BP1 levels (Figure 6(f-h)). Of note, the IPA precursor IAA, was not capable of blocking the phosphorylation of mTOR, S6 and 4E-BP1 (Figure 6(f-h)). Interestingly, in the absence of AhR signaling, the IL-17 expression was lower as previously desribed.^47^ However, the IPA-mediated effects on Th17 cells were not primarily AhR-dependent (Figure 6(i)). Finally, we observed that the supernatant derived from *C. sporogenes*, which was orally administered to SPF mice, partially reduced the severity of DSS-induced colitis as shown by less weight loss and reduced migration of pro-inflammatory T cells into the colon (Figures S4E, F). In conclusion, our data suggest that the microbial metabolites SCFAs, BCFAs, and IPA act cooperatively on the mucosal immune system to ameliorate intestinal inflammation after DSS exposure.

## Discussion

Commensals have evolved a huge functional diversity of enzyme complexes that enable them to catalyze various chemical reactions and synthesize microbial products that may have a therapeutic potential for the host. By metabolizing dietary carbohydrates and proteins, commensal bacteria can generate small molecules such as SCFAs, BCFAs and modified amino acids (e.g. microbial tryptophan catabolites), which are involved in the cross-talk between bacteria, enterocytes, antigen-presenting cells and lymphocytes in the gut.^48,49^ A better understanding and characterization of such molecules might provide insights into the development of novel microbiome-based therapeutic strategies. Alteration or reduction of the microbiome composition often leads to the dysfunction of the intestinal immune barrier and the development of chronic inflammation.^10^ Previous studies have demonstrated that some individual commensals are capable of modulating the transcriptional program and epigenetic status of epithelial and immune cells resulting in protection against colitis. Mice mono-colonized with *Bacteroides fragilis* were protected from DSS-induced colitis due to modulation of the immune system and dampening of inflammatory responses in the colon.^50^ This commensal is known to produce small molecules such as the capsular carbohydrate polysaccharide A and SCFAs that promote differentiation of Tregs and regulatory B cells (Bregs).^51,52^ Similarly, *Enterobacter ludwigii* was reported to exhibit protective effects during the intestinal inflammation through choline-mediated immune responses.^53^ In contrast, some bacteria such as *Porphyromonas gingivalis* can aggravate colitis by suppressing local linoleic acid production in the gut lumen. The blockade of LA metabolism resulted in reduced Treg activity and enhanced polarization of T cells toward a pathogenic Th17 phenotype.^54^

In recent years, several studies revealed a modulating role for microbiome-derived molecules in regulating innate and adaptive immunity.^26,55^ Upon stimulation with microbial-associated molecules such as butyrate and propionate, T cells upregulate *Foxp3* expression and induce secretion of anti-inflammatory cytokine IL-10 to restrict pro-inflammatory immune responses.^13,56^ Recently, we found that the SCFA pentanoate, specifically produced by the low-abundant commensal *Megasphaera massiliensis*, protected mice from experimental autoimmune encephalomyelitis.^45^ In our current study, we investigated whether the human commensal *C. sporogenes*, previously shown to promote neuroprotective effects,^57^ has an impact on the onset and development of colonic inflammation. We were surprised to see that some metabolites such as IPA and BCFAs were extensively secreted into the gut lumen and were even circulating in the blood. Particularly IPA, which activates the pregnane X receptor and aryl hydrocarbon receptor,^58,59^ was present at high concentrations in the gut and bloodstream. *C. sporogenes* completely protected mice from epithelial injury and infiltration of pro-inflammatory immune cells into the colonic mucosa, preventing the mortality seen in GF animals and ameliorating colitis following oral DSS administration. This observation is consistent with a metabolomic profiling approach by Alexeev *et al*., which showed that animals and patients with active colitis had a selective depletion of indole and indole-derived metabolites, in particular IPA.^60^ By colonizing mice with *C. sporogenes* we were able to restore IPA levels, promoting survival and alleviation of colitis. Pathogenic Th17 cells are well known for their role in promoting colitis as mice deficient for IL-17A do not show signs of inflammation following DSS treatment.^61^ Notably, IPA was able to suppress the IL-17A production by Th17 cells, which likely contributed to the protection against colonic inflammation.

**Figure 7.**
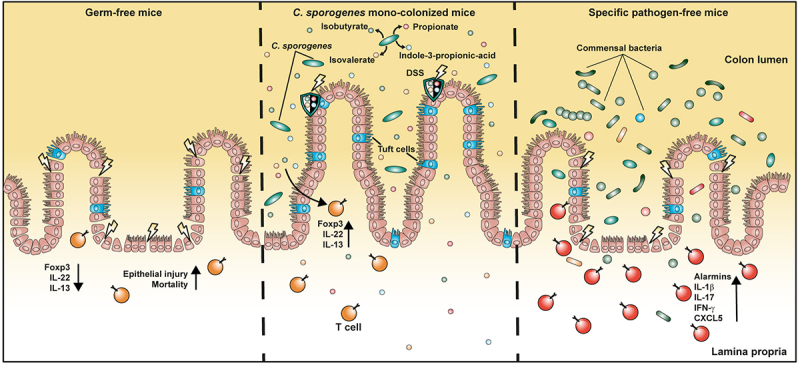
A schematic overview of the impact of *C. sporogenes* on the course of intestinal inflammation.

We observed an expansion of the tuft cell lineage in CS mice when compared to GF and SPF mice, which was most prominent in the colon, but also present to a lesser extent in the small intestine. CS mice also had the highest proportion of transcriptionally active tuft cells. Of note, a persistent higher abundance of colonic tuft cells was a feature of CS mice after DSS-induced colitis. At the transcriptional level, colonic tuft cells expressed the SCFA receptor, *Ffar3*, rendering these cells receptive to microbial metabolites, e.g. propionate and IPA. Indeed, in a mouse model of obesity, administration of IPA was found to be beneficial and reduced metabolic dysfunction, most likely via expansion of the tuft cell lineage and increased secretion of IL-25.^62^ Recently, chemosensory tuft cells were described as a protective mucosal cell type following inflammation in the human and mouse gut.^63^ Disturbances in tuft cell function exacerbated colitis in mice,^17,64^ and decreased numbers of ileal and colonic tuft cells were a feature of human patients suffering from intestinal inflammation.^65,66^ In contrast, expansion of the tuft cell lineage was associated with suppressed inflammation in a mouse model.^65,67^ A previous study demonstrated that IL-25 downregulated the Th1/Th17 immune response in IBD patients,^68^ which is consistent with our novel data in CS mice. Therefore, we hypothesize that the *C. sporogenes*-derived metabolites SCFAs and BCFAs promote expansion of the tuft cell lineage and activation via the SCFA-receptor Gpr41 (also known as FFAR3), which then leads to increased regeneration of the intestinal epithelium and suppression of inflammatory responses in the gut. In addition, both BCFAs and SCFAs produced by *C. sporogenes* increase the secretion of IL-22, a key protective factor that supports intestinal epithelial cell integrity (Figure 7). In conclusion, we suggest that *C. sporogenes* should be further therapeutically exploited as a probiotic bacterium to better understand the beneficial effects of its metabolites, which may be able to ameliorate disease progression in IBD patients.

## Supplementary Material

Supplemental Material

## Data Availability

Sequencing data were deposited at NCBI GEO under accession numbers GSE193358 (T cells) and GSE264024 (colon tissue). Any additional information or data that supports the findings of this study are available from the corresponding author upon reasonable request.

## References

[1] Graham DB, Xavier RJ. Conditioning of the immune system by the microbiome. Trends In Immunol. 2023;44(7):499–22. doi:10.1016/j.it.2023.05.002. 37236891

[2] Belkaid Y, Harrison OJ. Homeostatic immunity and the microbiota. Immunity. 2017;46(4):562–576. doi:10.1016/j.immuni.2017.04.008. 28423337 PMC5604871

[3] Atarashi K, Tanoue T, Shima T, Imaoka A, Kuwahara T, Momose Y, Cheng G, Yamasaki S, Saito T, Ohba Y, et al. Induction of colonic regulatory T cells by indigenous Clostridium species. Science. 2011;331(6015):337–341. doi:10.1126/science.1198469. 21205640 PMC3969237

[4] Takeuchi T, Miyauchi E, Kanaya T, Kato T, Nakanishi Y, Watanabe T, Kitami T, Taida T, Sasaki T, Negishi H, et al. Acetate differentially regulates IgA reactivity to commensal bacteria. Nature. 2021;595(7868):560–564. doi:10.1038/s41586-021-03727-5. 34262176

[5] Lo BC, Chen GY, Nunez G, Caruso R. Gut microbiota and systemic immunity in health and disease. Int Immunol. 2021;33(4):197–209. doi:10.1093/intimm/dxaa079. 33367688 PMC8011437

[6] Zheng D, Liwinski T, Elinav E. Interaction between microbiota and immunity in health and disease. Cell Res. 2020;30(6):492–506. doi:10.1038/s41422-020-0332-7. 32433595 PMC7264227

[7] Blumberg R, Powrie F. Microbiota, disease, and back to health: a metastable journey. Sci Transl Med. 2012;4(137):137rv137. doi:10.1126/scitranslmed.3004184. PMC5020897 22674557

[8] Wong SH, Yu J. Gut microbiota in colorectal cancer: mechanisms of action and clinical applications. Nat Rev Gastroenterol Hepatol. 2019;16(11):690–704. doi:10.1038/s41575-019-0209-8. 31554963

[9] Maloy KJ, Powrie F. Intestinal homeostasis and its breakdown in inflammatory bowel disease. Nature. 2011;474(7351):298–306. doi:10.1038/nature10208. 21677746

[10] Pisani A, Rausch P, Bang C, Ellul S, Tabone T, Marantidis Cordina C, Zahra G, Franke A, Ellul P, et al. Dysbiosis in the gut microbiota in patients with inflammatory bowel disease during remission. Microbiol Spectr. 2022;10(3):e0061622. doi:10.1128/spectrum.00616-22. 35532243 PMC9241752

[11] Visekruna A, Hartmann S, Sillke YR, Glauben R, Fischer F, Raifer H, Mollenkopf H, Bertrams W, Schmeck B, Klein M, et al. Intestinal development and homeostasis require activation and apoptosis of diet-reactive T cells. The J Clin Invest. 2019;129(5):1972–1983. doi:10.1172/JCI98929. 30939122 PMC6486345

[12] Macpherson AJ, Yilmaz B, Limenitakis JP, Ganal-Vonarburg SC. IgA function in relation to the intestinal microbiota. Annu Rev Immunol. 2018;36(1):359–381. doi:10.1146/annurev-immunol-042617-053238. 29400985

[13] Smith PM, Howitt MR, Panikov N, Michaud M, Gallini CA, Bohlooly-Y M, Glickman JN, Garrett WS. The microbial metabolites, short-chain fatty acids, regulate colonic treg cell homeostasis. Science. 2013;341(6145):569–573. doi:10.1126/science.1241165. 23828891 PMC3807819

[14] Ivanov II, Ivanov II, Atarashi K, Manel N, Brodie EL, Shima T, Karaoz U, Wei D, Goldfarb KC, Santee CA, et al. Induction of intestinal Th17 cells by segmented filamentous bacteria. Cell. 2009;139(3):485–498. doi:10.1016/j.cell.2009.09.033. 19836068 PMC2796826

[15] Zindl CL, Witte SJ, Laufer VA, Gao M, Yue Z, Janowski KM, Cai B, Frey BF, Silberger DJ, Harbour SN, et al. A nonredundant role for T cell-derived interleukin 22 in antibacterial defense of colonic crypts. Immunity. 2022;55(3):494–511 e411. doi:10.1016/j.immuni.2022.02.003. 35263568 PMC9126440

[16] Sugimoto K, Ogawa A, Mizoguchi E, Shimomura Y, Andoh A, Bhan AK, Blumberg RS, Xavier RJ, Mizoguchi A. IL-22 ameliorates intestinal inflammation in a mouse model of ulcerative colitis. J Clin Invest. 2008;118:534–544. doi:10.1172/JCI33194. 18172556 PMC2157567

[17] Qu D, Weygant N, May R, Chandrakesan P, Madhoun M, Ali N, Sureban SM, An G, Schlosser MJ, Houchen CW, et al. Ablation of doublecortin-like kinase 1 in the colonic epithelium exacerbates dextran sulfate sodium-induced colitis. PLOS ONE. 2015;10(8):e0134212. doi:10.1371/journal.pone.0134212. 26285154 PMC4540568

[18] Yang W, Yu T, Huang X, Bilotta AJ, Xu L, Lu Y, Sun J, Pan F, Zhou J, Zhang W, et al. Intestinal microbiota-derived short-chain fatty acids regulation of immune cell IL-22 production and gut immunity. Nat Commun. 2020;11(1):4457. doi:10.1038/s41467-020-18262-6. 32901017 PMC7478978

[19] Keshavarz M, Faraj Tabrizi S, Ruppert A-L, Pfeil U, Schreiber Y, Klein J, Brandenburger I, Lochnit G, Bhushan S, Perniss A, et al. Cysteinyl leukotrienes and acetylcholine are biliary tuft cell cotransmitters. Sci Immunol. 2022;7(69):eabf6734. doi:10.1126/sciimmunol.abf6734. 35245090

[20] Coutry N, Nguyen, J, Soualhi, S, Gerbe, F, Meslier, V, Dardalhon, V, Almeida, M, Quinquis, B, Thirion, F, Herbert, F, et al. Cross talk between Paneth and tuft cells drives dysbiosis and inflammation in the gut mucosa. Proceedings of the National Academy of Sciences of the United States of America; 2023 Jun 20; USA. Vol. 120. 2023. p. e2219431120. doi:10.1073/pnas.2219431120. PMC10288547 37307458

[21] Schneider C, O’Leary CE, von Moltke J, Liang H-E, Ang QY, Turnbaugh PJ, Radhakrishnan S, Pellizzon M, Ma A, Locksley RM, et al. A metabolite-triggered tuft cell-ILC2 circuit drives small intestinal remodeling. Cell. 2018;174(2):271–284 e214. doi:10.1016/j.cell.2018.05.014. 29887373 PMC6046262

[22] Nadjsombati MS, McGinty JW, Lyons-Cohen MR, Jaffe JB, DiPeso L, Schneider C, Miller CN, Pollack JL, Nagana Gowda GA, Fontana MF, et al. Detection of succinate by intestinal tuft cells triggers a type 2 innate immune circuit. Immunity. 2018;49(1):33–41 e37. doi:10.1016/j.immuni.2018.06.016. 30021144 PMC6084797

[23] Lei W, Ren, W, Ohmoto, M, Urban Jr, JF, Matsumoto, I, Margolskee, RF, Jiang, P. Activation of intestinal tuft cell-expressed Sucnr1 triggers type 2 immunity in the mouse small intestine. Proceedings of the National Academy of Sciences of the United States of America; 22 May 2018; USA. Vol. 115. 2018. p. 5552–5557 . doi: 10.1073/pnas.1720758115. PMC6003470 29735652

[24] Luu M, Visekruna A. Microbial metabolites: novel therapeutic tools for boosting cancer therapies. Trends Cell Biol. 2021;31(11):873–875. doi:10.1016/j.tcb.2021.08.005. 34538658

[25] Agus A, Clement K, Sokol H. Gut microbiota-derived metabolites as central regulators in metabolic disorders. Gut. 2021;70(6):1174–1182. doi:10.1136/gutjnl-2020-323071. 33272977 PMC8108286

[26] Michaudel C, Sokol H. The gut microbiota at the service of immunometabolism. Cell Metab. 2020;32(4):514–523. doi:10.1016/j.cmet.2020.09.004. 32946809

[27] Dodd D, Spitzer MH, Van Treuren W, Merrill BD, Hryckowian AJ, Higginbottom SK, Le A, Cowan TM, Nolan GP, Fischbach MA, et al. A gut bacterial pathway metabolizes aromatic amino acids into nine circulating metabolites. Nature. 2017;551(7682):648–652. doi:10.1038/nature24661. 29168502 PMC5850949

[28] Jiang H, Chen C, Gao J. Extensive summary of the important roles of indole propionic acid, a gut microbial metabolite in host health and disease. Nutrients. 2022;15(1):151. doi:10.3390/nu15010151. 36615808 PMC9824871

[29] von Engelhardt J, Eliava M, Meyer AH, Rozov A, Monyer H. Functional characterization of intrinsic cholinergic interneurons in the cortex. J Neurosci. 2007;27(21):5633–5642. doi:10.1523/JNEUROSCI.4647-06.2007. 17522308 PMC6672773

[30] Lotti C, Rubert J, Fava F, Tuohy K, Mattivi F, Vrhovsek U. Development of a fast and cost-effective gas chromatography–mass spectrometry method for the quantification of short-chain and medium-chain fatty acids in human biofluids. Anal Bioanal Chem. 2017;409(23):5555–5567. doi:10.1007/s00216-017-0493-5. 28717897

[31] Romero R, Zarzycka A, Preussner M, Fischer F, Hain T, Herrmann J-P, Roth K, Keber CU, Suryamohan K, Raifer H, et al. Selected commensals educate the intestinal vascular and immune system for immunocompetence. Microbiome. 2022;10(1):158. doi:10.1186/s40168-022-01353-5. 36171625 PMC9520927

[32] Erben U, Loddenkemper C, Doerfel K, Spieckermann S, Haller D, Heimesaat MM, Zeitz M, Siegmund B, Kühl AA. A guide to histomorphological evaluation of intestinal inflammation in mouse models. Int J Clin Exp Pathol. 2014;7(8):4557–4576. 25197329 PMC4152019

[33] Schutz B, Jurastow I, Bader S, Ringer C, von Engelhardt J, Chubanov V, Gudermann T, Diener M, Kummer W, Krasteva-Christ G, et al. Chemical coding and chemosensory properties of cholinergic brush cells in the mouse gastrointestinal and biliary tract. Front Physiol. 2015;6:87. doi:10.3389/fphys.2015.00087. 25852573 PMC4371653

[34] Schutz B, Ruppert A-L, Strobel O, Lazarus M, Urade Y, Büchler MW, Weihe E. Distribution pattern and molecular signature of cholinergic tuft cells in human gastro-intestinal and pancreatic-biliary tract. Sci Rep. 2019;9(1):17466. doi:10.1038/s41598-019-53997-3. 31767912 PMC6877571

[35] Li TT, et al. Microbiota metabolism of intestinal amino acids impacts host nutrient homeostasis and physiology. Cell Host & Microbe. 2024;32:661–675 e610. doi:10.1016/j.chom.2024.04.004. 38657606 PMC11636940

[36] Luu M, Visekruna A. Short-chain fatty acids: bacterial messengers modulating the immunometabolism of T cells. Eur J Immunol. 2019;49(6):842–848. doi:10.1002/eji.201848009. 31054154

[37] Liu Y, Chen H, Van Treuren W, Hou B-H, Higginbottom SK, Dodd D. Clostridium sporogenes uses reductive Stickland metabolism in the gut to generate ATP and produce circulating metabolites. Nat Microbiol. 2022;7(5):695–706. doi:10.1038/s41564-022-01109-9. 35505245 PMC9089323

[38] Strine MS, Wilen CB, Lazear HM. Tuft cells are key mediators of interkingdom interactions at mucosal barrier surfaces. PLOS Pathog. 2022;18(3):e1010318. doi:10.1371/journal.ppat.1010318. 35271673 PMC8912186

[39] Silverman JB, Vega PN, Tyska MJ, Lau KS. Intestinal tuft cells: morphology, function, and implications for human health. Annu Rev Physiol. 2024;86(1):479–504. doi:10.1146/annurev-physiol-042022-030310. 37863104 PMC11193883

[40] Yamashita J, Ohmoto M, Yamaguchi T, Matsumoto I, Hirota J, Ishimaru Y. Skn-1a/Pou2f3 functions as a master regulator to generate Trpm5-expressing chemosensory cells in mice. PLOS ONE. 2017;12(12):e0189340. doi:10.1371/journal.pone.0189340. 29216297 PMC5720759

[41] Park SE, Lee D, Jeong JW, Lee S-H, Park SJ, Ryu J, Oh SK, Yang H, Fang S, Kim S, et al. Gut epithelial inositol polyphosphate multikinase alleviates experimental colitis via governing tuft cell homeostasis. Cell Mol Gastroenterol Hepatol. 2022;14(6):1235–1256. doi:10.1016/j.jcmgh.2022.08.004. 35988719 PMC9579329

[42] Atarashi K, Tanoue T, Oshima K, Suda W, Nagano Y, Nishikawa H, Fukuda S, Saito T, Narushima S, Hase K, et al. Treg induction by a rationally selected mixture of clostridia strains from the human microbiota. Nature. 2013;500(7461):232–236. doi:10.1038/nature12331. 23842501

[43] Steele SP, Melchor SJ, Petri WA Jr. Tuft cells: new players in colitis. Trends Mol Med. 2016;22(11):921–924. doi:10.1016/j.molmed.2016.09.005. 27717671 PMC5159242

[44] Hernandez-Chirlaque C, Aranda CJ, Ocón B, Capitán-Cañadas F, Ortega-González M, Carrero JJ, Suárez MD, Zarzuelo A, Sánchez de Medina F, Martínez-Augustin O, et al. Germ-free and antibiotic-treated mice are highly susceptible to epithelial injury in DSS colitis. J Crohns Colitis. 2016;10(11):1324–1335. doi:10.1093/ecco-jcc/jjw096. 27117829

[45] Luu M, Pautz S, Kohl V, Singh R, Romero R, Lucas S, Hofmann J, Raifer H, Vachharajani N, Carrascosa LC, et al. The short-chain fatty acid pentanoate suppresses autoimmunity by modulating the metabolic-epigenetic crosstalk in lymphocytes. Nat Commun. 2019;10(1):760. doi:10.1038/s41467-019-08711-2. 30770822 PMC6377655

[46] Park SH, Cho G, Park SG. Nf-κB activation in T helper 17 cell differentiation. Immune Network. 2014;14(1):14–20. doi:10.4110/in.2014.14.1.14. 24605076 PMC3942503

[47] Stockinger B, Di Meglio P, Gialitakis M, Duarte JH. The aryl hydrocarbon receptor: multitasking in the immune system. Annu Rev Immunol. 2014;32(1):403–432. doi:10.1146/annurev-immunol-032713-120245. 24655296

[48] Koh A, De Vadder F, Kovatcheva-Datchary P, Backhed F. From dietary fiber to Host physiology: short-chain fatty acids as key bacterial metabolites. Cell. 2016;165(6):1332–1345. doi:10.1016/j.cell.2016.05.041. 27259147

[49] Shine EE, Crawford JM. Molecules from the microbiome. Annu Rev Biochem. 2021;90(1):789–815. doi:10.1146/annurev-biochem-080320-115307. 33770448

[50] Chiu CC, Ching Y-H, Wang Y-C, Liu J-Y, Li Y-P, Huang Y-T, Chuang H-L. Monocolonization of germ-free mice with bacteroides fragilis protects against dextran sulfate sodium-induced acute colitis. Biomed Res Int. 2014;2014:1–9. doi:10.1155/2014/675786. PMC4058166 24971344

[51] Round JL, Lee SM, Li J, Tran G, Jabri B, Chatila TA, Mazmanian SK. The Toll-like receptor 2 pathway establishes colonization by a commensal of the human microbiota. Science. 2011;332(6032):974–977. doi:10.1126/science.1206095. 21512004 PMC3164325

[52] Ramakrishna C, Kujawski M, Chu H, Li L, Mazmanian SK, Cantin EM. Bacteroides fragilis polysaccharide a induces IL-10 secreting B and T cells that prevent viral encephalitis. Nat Commun. 2019;10(1):2153. doi:10.1038/s41467-019-09884-6. 31089128 PMC6517419

[53] Li Q, Sun X, Yu K, Lv J, Miao C, Yang J, Wang S, Fu Z, Sun Y, Zhang H, et al. Enterobacter ludwigii protects dss-induced colitis through choline-mediated immune tolerance. Cell Rep. 2022;40(9):111308. doi:10.1016/j.celrep.2022.111308. 36044853

[54] Jia L, Jiang Y, Wu L, Fu J, Du J, Luo Z, Guo L, Xu J, Liu Y. Porphyromonas gingivalis aggravates colitis via a gut microbiota-linoleic acid metabolism-Th17/Treg cell balance axis. Nat Commun. 2024;15(1):1617. doi:10.1038/s41467-024-45473-y. 38388542 PMC10883948

[55] Levy M, Thaiss CA, Elinav E. Metabolites: messengers between the microbiota and the immune system. Genes Dev. 2016;30(14):1589–1597. doi:10.1101/gad.284091.116. 27474437 PMC4973288

[56] Arpaia N, Campbell C, Fan X, Dikiy S, van der Veeken J, deRoos P, Liu H, Cross JR, Pfeffer K, Coffer PJ, et al. Metabolites produced by commensal bacteria promote peripheral regulatory T-cell generation. Nature. 2013;504(7480):451–455. doi:10.1038/nature12726. 24226773 PMC3869884

[57] Serger E, Luengo-Gutierrez L, Chadwick JS, Kong G, Zhou L, Crawford G, Danzi MC, Myridakis A, Brandis A, Bello AT, et al. The gut metabolite indole-3 propionate promotes nerve regeneration and repair. Nature. 2022;607(7919):585–592. doi:10.1038/s41586-022-04884-x. 35732737

[58] Venkatesh M, Mukherjee S, Wang H, Li H, Sun K, Benechet A, Qiu Z, Maher L, Redinbo M, Phillips R, et al. Symbiotic bacterial metabolites regulate gastrointestinal barrier function via the xenobiotic sensor PXR and Toll-like receptor 4. Immunity. 2014;41(2):296–310. doi:10.1016/j.immuni.2014.06.014. 25065623 PMC4142105

[59] Roager HM, Licht TR. Microbial tryptophan catabolites in health and disease. Nat Commun. 2018;9(1):3294. doi:10.1038/s41467-018-05470-4. 30120222 PMC6098093

[60] Alexeev EE, Lanis JM, Kao DJ, Campbell EL, Kelly CJ, Battista KD, Gerich ME, Jenkins BR, Walk ST, Kominsky DJ, et al. Microbiota-derived indole metabolites promote human and murine intestinal homeostasis through regulation of interleukin-10 receptor. The Am J Pathol. 2018;188(5):1183–1194. doi:10.1016/j.ajpath.2018.01.011. 29454749 PMC5906738

[61] Vachharajani N, Joeris T, Luu M, Hartmann S, Pautz S, Jenike E, Pantazis G, Prinz I, Hofer MJ, Steinhoff U, et al. Prevention of colitis-associated cancer by selective targeting of immunoproteasome subunit LMP7. Oncotarget. 2017;8(31):50447–50459. doi:10.18632/oncotarget.14579. 28881574 PMC5584149

[62] Chen L, Yang Y, Sun S, Xie Y, Pan C, Li M, Li C, Liu Y, Xu Z, Liu W, et al. Indolepropionic acid reduces obesity-induced metabolic dysfunction through colonic barrier restoration mediated via tuft cell-derived IL-25. The FEBS J. 2022;289(19):5985–6004. doi:10.1111/febs.16470. 35509122

[63] Schneider C, O’Leary CE, Locksley RM. Regulation of immune responses by tuft cells. Nat Rev Immunol. 2019;19(9):584–593. doi:10.1038/s41577-019-0176-x. 31114038 PMC8331098

[64] Yi J, Bergstrom K, Fu J, Shan X, McDaniel JM, McGee S, Qu D, Houchen CW, Liu X, Xia L, et al. Dclk1 in tuft cells promotes inflammation-driven epithelial restitution and mitigates chronic colitis. Cell Death Differ. 2019;26(9):1656–1669. doi:10.1038/s41418-018-0237-x. 30478383 PMC6748088

[65] Banerjee A, Herring CA, Chen B, Kim H, Simmons AJ, Southard-Smith AN, Allaman MM, White JR, Macedonia MC, Mckinley ET, et al. Succinate produced by intestinal microbes promotes specification of tuft cells to suppress ileal inflammation. Gastroenterology. 2020;159(6):2101–2115, e2105. doi:10.1053/j.gastro.2020.08.029. 32828819 PMC7725941

[66] Kjaergaard S, Jensen TSR, Feddersen UR, Bindslev N, Grunddal KV, Poulsen SS, Rasmussen HB, Budtz-Jørgensen E, Berner-Hansen M. Decreased number of colonic tuft cells in quiescent ulcerative colitis patients. Eur J Gastroenterol Hepatol. 2021;33(6):817–824. doi:10.1097/MEG.0000000000001959. 33079783 PMC8083166

[67] Coutry N, Gasmi I, Herbert F, Jay P. Mechanisms of intestinal dysbiosis: new insights into tuft cell functions. Gut Microbes. 2024;16(1):2379624. doi:10.1080/19490976.2024.2379624. 39042424 PMC11268228

[68] Su J, Chen T, Ji X-Y, Liu C, Yadav PK, Wu R, Yang P, Liu Z. IL-25 Downregulates Th1/Th17 immune response in an IL-10–dependent manner in inflammatory bowel disease. Inflamm Bowel Dis. 2013;19(4):720–728. doi:10.1097/MIB.0b013e3182802a76. 23429464

