# Supplementary material for: Clostridium sporogenes-derived metabolites protect mice against colonic inflammation
Source: Gut Microbes. 2024 Oct 14;16(1):2412669. doi: 10.1080/19490976.2024.2412669 (PMC11485882; doi:10.1080/19490976.2024.2412669)
Supplement: Supplemental Material [file KGMI_A_2412669_SM5819.zip › REVISION_Supplement_Krause_et_al_clean.docx]

***Clostridium sporogenes*-derived metabolites protect mice against colonic inflammation**

Felix F Krause^1^, Kira I Mangold^1^, Anna-Lena Ruppert^2^, Hanna Leister^1^, Anne Hellhund-Zingel^1^, Aleksandra Lopez Krol^1^, Jelena Pesek^3^, Bernhard Watzer^3^, Sarah Winterberg^2^, Hartmann Raifer^4^, Kai Binder^1^, Ralf Kinscherf^2^, Alesia Walker^5^, Wolfgang A Nockher^3^, R Verena Taudte^3^, Wilhelm Bertrams^6^, Bernd Schmeck^6,7,8^, Anja A Kühl^9^, Britta Siegmund^10^, Rossana Romero^1^, Maik Luu^11^, Stephan Göttig^12^, Isabelle Bekeredjian-Ding^1^, Ulrich Steinhoff^1^, Burkhard Schütz^2,13^ and Alexander Visekruna^1,13^

^1^Institute for Medical Microbiology and Hygiene, Philipps-University, Marburg, Germany.
^2^Institute of Anatomy and Cell Biology, Philipps-University, Marburg, Germany.

^3^Core Facility for Metabolomics, Department of Medicine, Philipps-University Marburg, Germany.

^4^Flow Cytometry Core Facility, Philipps-University, Marburg, Germany.

^5^Research Unit Analytical BioGeoChemistry, Helmholtz Zentrum München, Neuherberg, Germany.

^6^Institute for Lung Research, Philipps-University, Marburg, Germany.

^7^Department for Respiratory and Critical Care Medicine, Philipps-University, Marburg, Germany.

^8^Member of the German Center for Lung Research (DZL/UGMLC,) and German Center for Infectious

Disease Research (DZIF), Marburg, Germany.

^9^iPATH.Berlin, Core Unit of Charité-Universitätsmedizin Berlin, Charité - Universitätsmedizin Berlin, corporate member of Freie Universität Berlin, Humboldt-Universität zu Berlin, and Berlin Institute of Health, Berlin, Germany.

^10^Department of Gastroenterology, Infectious Diseases and Rheumatology, Charité - Universitätsmedizin Berlin, corporate member of Freie Universität Berlin, Humboldt-Universität zu Berlin, Campus Benjamin Franklin, Berlin, Germany.

^11^Lehrstuhl für Zelluläre Immuntherapie, Medizinische Klinik und Poliklinik II, Universitätsklinikum Würzburg, Würzburg, Germany.

^12^Goethe University Frankfurt, University Hospital, Institute of Medical Microbiology and Infection Control, Frankfurt am Main, Germany.

^13^These authors jointly supervised this work.

Correspondence: alexander.visekruna@staff.uni-marburg.de


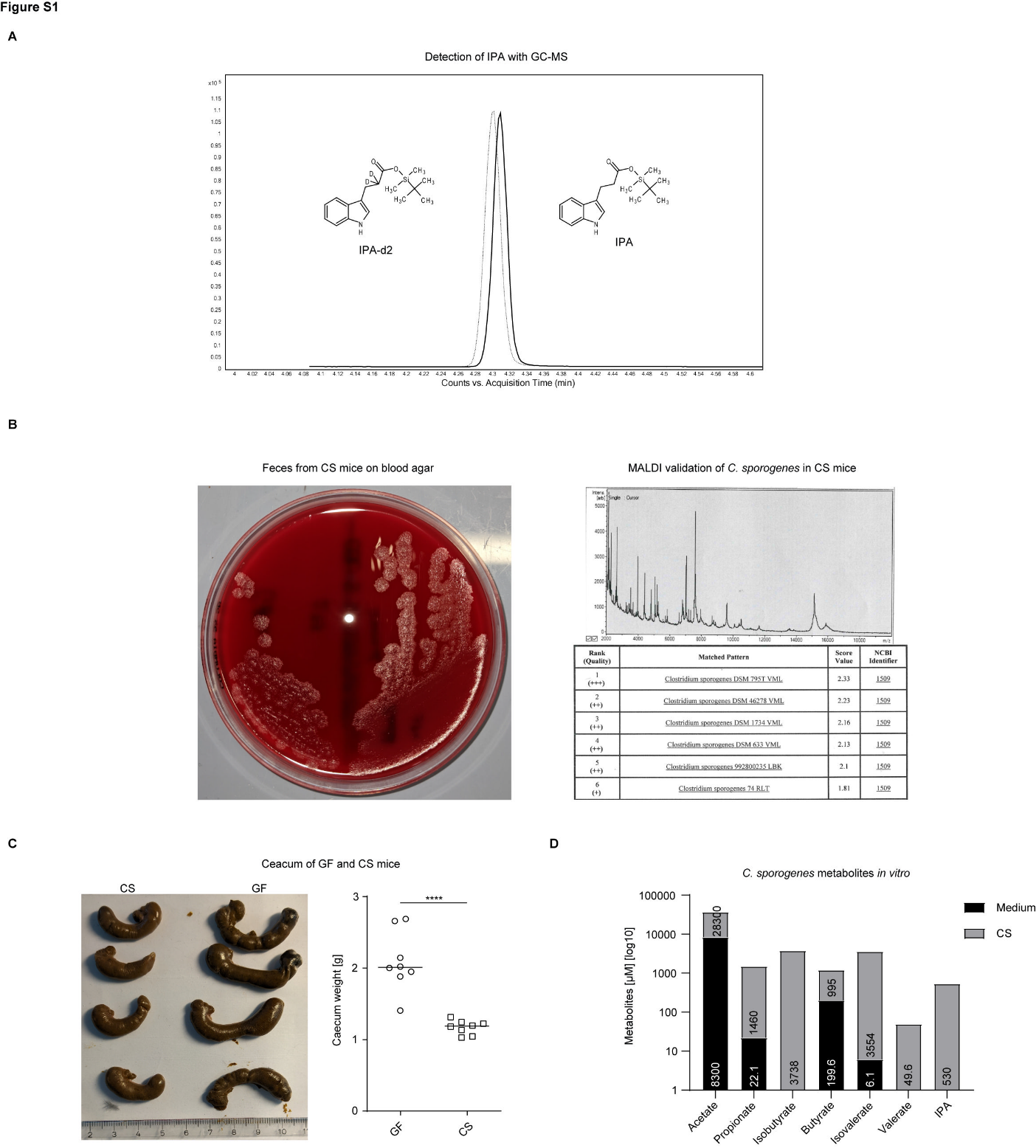


**Supplementary Figure 1.** **Caecum size in *C. sporogenes* mono-colonized mice and *in vitro* metabolites production.**

(**A**) Typical chromatogram showing the extracted ion traces of IPA-d2 (m/z 305; dotted lines; left) and IPA (m/z 303; bold lines; right) including the structures of the corresponding TBDMS derivatives. (**B**) Left: Faeces from CS mice were plated on blood agar plates to verify mono-colonisation. Right: Colonies were analysed by MALDI-TOF-MS to confirm identity. Shown are the MALDI-TOF MS spectrum and the top matching species (all *C. sporogenes*). (**C**) Caecum size and weight of GF and CS mice. GF mice were colonized with *C. sporogenes* at the age of 1 month and analyzed after 2 months of colonization (*n*= 8). Statistical analysis was done with one-way ANOVA with *****p* < 0.0001. Data are presented as means. (**D**) Levels of SCFAs, BCFAs and IPA produced by *C. sporogenes* *in vitro*. *C. sporogenes* was grown in BHI medium for 3 days in an anaerobic environment at 37°C and metabolites were measured via GC-MS.


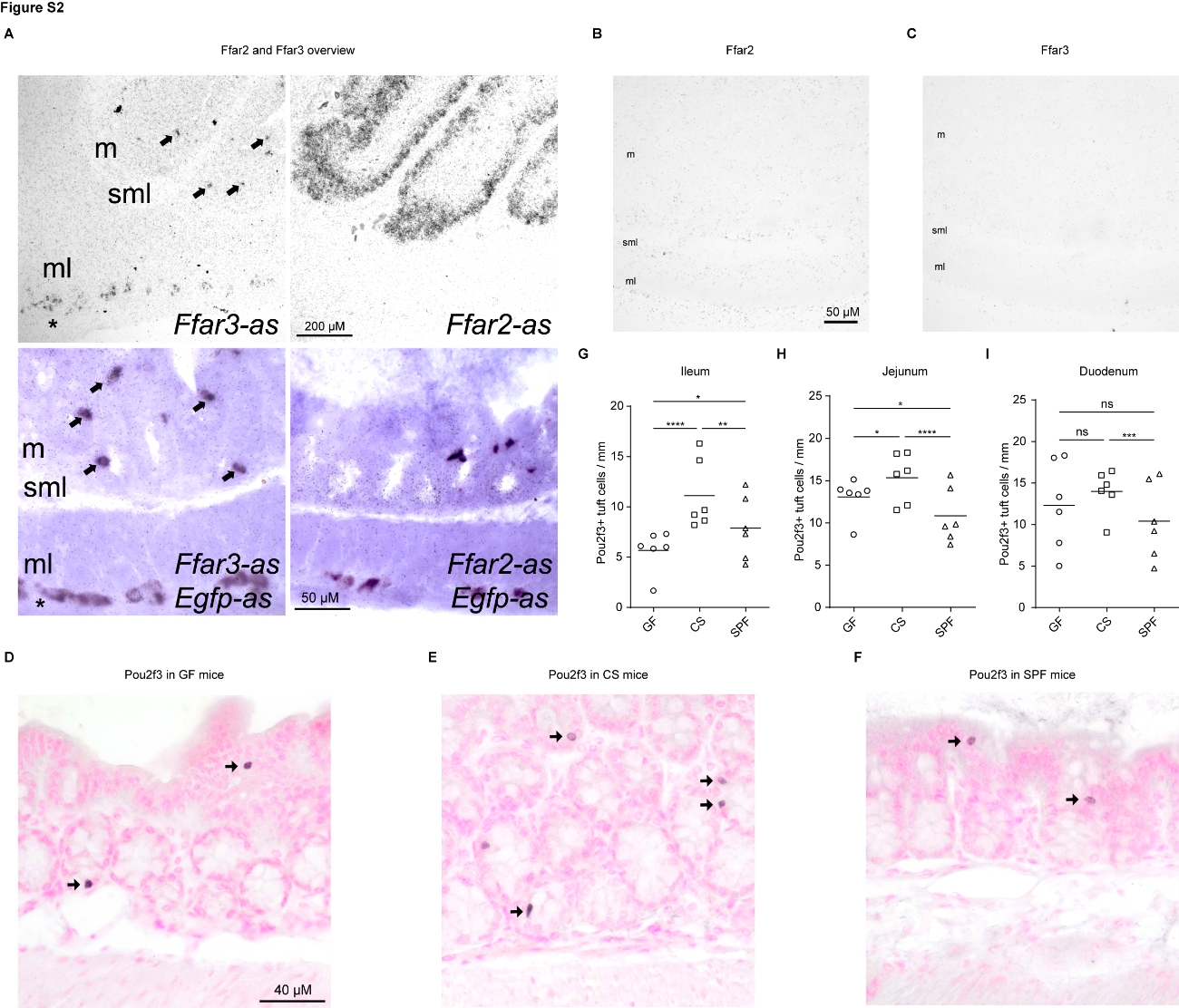


**Supplementary Figure 2. Pou2f3 and Ffar2/3 detection in mice.**

(**A**) Top: Detection of *Ffar2* and *Ffar3* mRNA in colonic tissue sections of C57BL/6 mice by *in situ* hybridization (ISH) using radioactive-labeled (S35) anti-sense (as) RNA probes. Bottom: mRNA detection of *Ffar2* and *Ffar3* by double-labeled ISH in colonic tuft cells (Egfpdig-labeled in Chat-EGFP mice indicated by arrows) (*n* = 5). (**B** and **C**) Sense riboprobes for (B) *Ffar2* and (C) *Ffar3* show no unspecific binding. m = mucosa; sml = submucosal layer; ml = muscle layer; * = presence of *Ffar3* mRNA in myenteric plexus neurons. (**D-F**) Representative detection of Pou2f3^+^ tuft cells (labeled by arrows) in the colon of (D) GF, (E) CS and (F) SPF mice. Tissue was counterstained with nuclear red. (**G-I**) The density of epithelial tuft cells in the (G) ileum, (H) jejunum, and (I) duodenum was analyzed in GF, CS, and SPF mice by Pou2f3 immunohistochemistry (*n* = 6). Statistical analysis was done with two-way ANOVA with Tukey post hoc test with **p* < 0.05; ***p* < 0.01; ****p* < 0.001; *****p* < 0.0001. The horizontal line represents the mean.


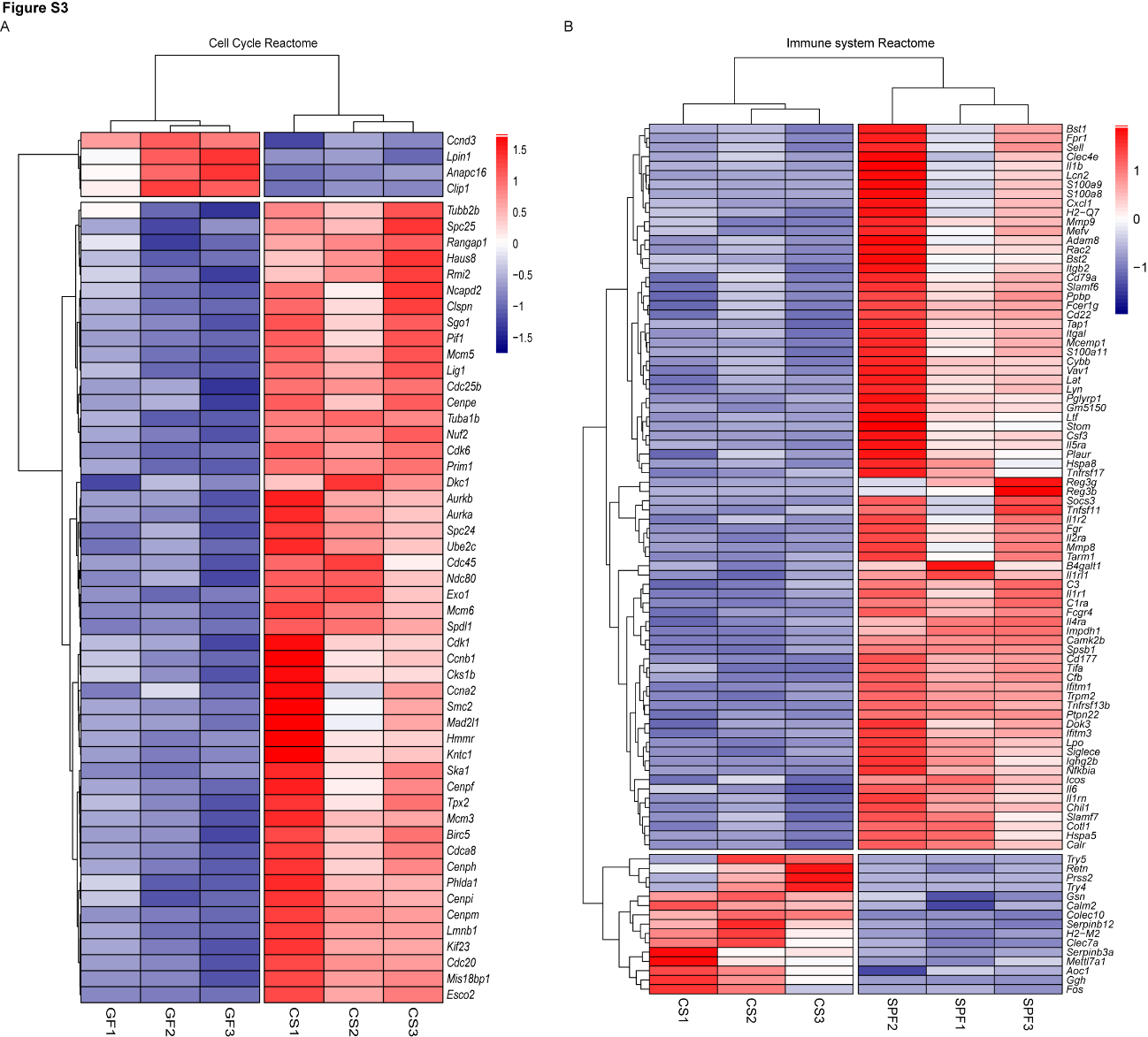


**Supplementary Figure 3**. **Analysis of differentially regulated genes in GF, CS and SPF mice.**

(**A** and **B**) GF, CS and SPF mice were treated with DSS (2.5%) in drinking water for 5 days and analyzed on day 7. Colon tissue was harvested, and RNA was extracted and sequenced (*n* = 3). Heat map displaying significantly changed genes in the KEGG pathways (A) “cell cycle” between GF and CS mice after DSS-induced colitis. (B) Heat map displaying significantly changed genes in the Reactome pathway “Immune system”, between CS and SPF mice after DSS-induced colitis. Expression changes of genes were considered significant with p adjusted value < 0.05. Transcripts per million (TPM) values were computed, z-score transformed and represented in heatmaps. Heatmaps were generated with the R package pheatmap v. 1.0.12 using Euclidean distances. Trees indicate hierarchical clustering (complete method).


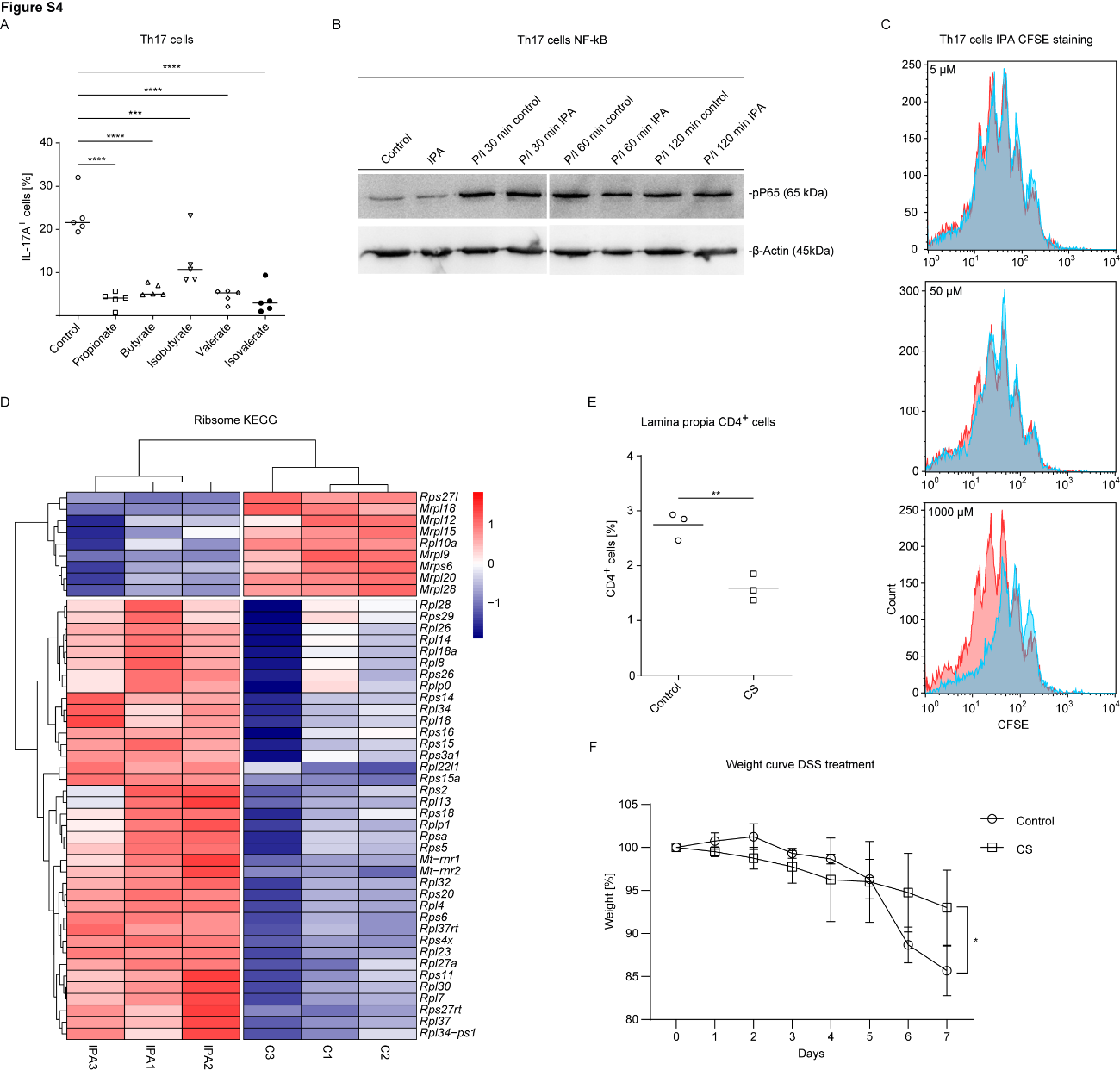


**Supplementary Figure 4. Gavage of *C. sporogenes*-derived supernatant improves DSS-induced colitis.**

(**A-C**) CD4^+^ T cells were isolated from two to four-month-old C57BL/6N mice and differentiated under Th17-polarizing conditions for 3 days. Cells were treated with different stimuli starting on day 0. (A) Flow cytometry analysis of Th17 cells showing the percentage of IL-17A^+^ cells after 3 days. Cells were treated with propionate, isobutyrate, valerate, isovalerate at 2 mM each and butyrate at 0.5 mM (*n* = 5). Statistical analysis was done with one-way ANOVA with ****p* < 0.001; *****p* < 0.0001. Horizontal lines represent the means. (B) Western blot of NF-κB p-p65 on Th17 cells treated with 50 µM IPA for 3 days and phorbol 12-myristate 13-acetate (PMA) / ionomycin (P/I) for indicated time points. β-actin was used as a loading control. (C) CFSE staining of Th17 cells treated with IPA (5, 50 and 1000 µM) for 3 days. The red area indicates control cells and the blue area IPA-treated cells. (**D**) Heatmap of RNAseq displaying all significantly (*p*adj. < 0.05) changed genes between control and IPA treated Th17 cells in the KEGG pathway “Ribosome”. (**E** and **F**) SPF mice were treated with DSS (2.5%) in drinking water for 5 days and analyzed on day 7. Mice were gavaged every second day with *C. sporogenes* supernatant, grown in BHI for 3 days in an anaerobic environment, or BHI medium as control. (E) Scatter plot showing the percentage of live CD4^+^ T cells in the colon lamina propria of DSS-treated mice (*n* = 3). (F) Weight curve of DSS-treated mice (*n* = 4). Statistical analysis was done with two-way ANOVA with **p* < 0.05. Data are presented as mean and error bars represent SD. Statistical analysis was done with one-way ANOVA with ***p* < 0.01. Horizontal lines represent the mean.

Supplementary Table 1. GC-MS temperature program for IPA measurement

| Rate/°C min^-1^ | Value/°C | Hold time/min | Run time/min |
| --- | --- | --- | --- |
|  | 220 | 1 | 1 |
| 15 | 320 | 4 | 9.67 |

Supplementary Table 2. GC-MS temperature program for SCFAs and BCFAs measurement

| Rate/°C min^-1^ | Value/°C | Hold time/min | Run time/min |
| --- | --- | --- | --- |
|  | 40 | 0 | 0 |
| 10 | 180 | 0 | 14 |
| 70 | 250 | 1 | 16 |

Supplementary Table 3. MRM transitions, collision energy values and retention times of the SCFAs and BCFAs for GC-MS method.

| Compound | C atoms | Precursor ion | Product ion | Collision energy/eV | Retention time/min |
| --- | --- | --- | --- | --- | --- |
| Acetic acid-d3 | C2 | 63 | 46 | 5 | 8.7 |
| Acetic acid | C2 | 60 | 45 | 10 | 8.7 |
| Propionic acid-d3 | C3 | 77 | 76 | 5 | 9.8 |
| Propionic acid | C3 | 74 | 73 | 5 | 9.8 |
| Isobutyric acid-d7 | C4 | 95 | 77 | 10 | 10.1 |
| Isobutyric acid | C4 | 88 | 73 | 5 | 10.2 |
| Butyric acid-d3 | C4 | 63 | 44 | 5 | 10.8 |
| Butyric acid | C4 | 60 | 42 | 10 | 10.9 |
| Isovaleric acid-d9 | C5 | 63 | 44 | 5 | 11.3 |
| Isovaleric acid | C5 | 60 | 42 | 10 | 11.4 |
| Valeric acid-d3 | C5 | 63 | 44 | 5 | 12.1 |
| Valeric acid | C5 | 60 | 42 | 10 | 12.2 |
| Caproic acid-d11 | C6 | 63 | 44 | 5 | 13 |
| Caproic acid | C6 | 60 | 42 | 10 | 13 |
